# Fusion-Derived Centromeres Reveal Satellite Repeat Remodeling in *Miscanthus sinensis*

**DOI:** 10.3390/plants15182855

**Published:** 2026-09-18

**Authors:** Qi Yang, Yu Zhang, Zhuo Wang, Zetian Cui, Liangchang Zhang, Fengzhen Li, Zehuai Yu

**Affiliations:** 1 College of Bioengineering, Hunan Polytechnic of Environment and Biology, Hengyang 421005, China; 2 National Engineering Research Center for Sugarcane, Key Laboratory of Sugarcane Biology and Genetic Breeding, Ministry of Agriculture and Rural Affairs, Fujian Agriculture and Forestry University, Fuzhou 350002, China; 3 Guangxi Key Laboratory for Sugarcane Biology, State Key Laboratory for Conservation and Utilization of Subtropical Agro-Bioresources, Guangxi University, Nanning 530004, China

**Keywords:** centromeres, FISH, *Miscanthus*, allotetraploid

## Abstract

Centromeres are essential for chromosome segregation, yet their repetitive nature makes them among the most difficult genomic regions to assemble and interpret. In polyploid plants, centromeres provide a unique record of chromosome evolution because centromeric repeats can be reshaped by polyploidization, subgenome differentiation and chromosomal rearrangement. Here, we combined low-coverage repeatome profiling, genome-wide mapping, fluorescence in situ hybridization (FISH), fiber-FISH and higher-order repeats (HORs) analysis to investigate centromere-associated repeats in *Miscanthus sinensis* (2n = 2*x* = 38). We identified MsSat1, a 137 bp satellite repeat representing approximately 2.1% of the genome, as a major centromere-associated satellite repeat. MsSat1 signals overlapped with the *Saccharum officinarum* centromeric retrotransposon probe and formed continuous tandem arrays on DNA fibers, supporting its association with centromeric domains. MsSat1-based clustering further recovered 13 related satellite variants, many of which showed strongly biased distribution between the A and B subgenomes. Among them, MsSat1-G displayed the most striking pattern, with strong B-subgenome enrichment and chromosome 7-specific localization. Detailed analysis revealed that MsSat1-G was embedded within an expanded MsSat1-rich domain on the fusion-derived chromosome 7, where it showed a more heterogeneous HORs architecture than the major MsSat1 array. These findings suggest that chromosome fusion was accompanied by local centromeric satellite amplification and repeat-architecture remodeling. Together, our study reveals that fusion-derived centromeres can preserve distinct satellite signatures and provides cytogenetic and genomic evidence that centromeric repeats record both subgenome differentiation and chromosome restructuring in *M. sinensis*.

## 1. Introduction

Centromeres are among the most essential chromosomal domains, ensuring accurate chromosome segregation and stable genome inheritance during cell division [1,2,3]. Yet, paradoxically, these essential domains have long remained among the darkest regions of eukaryotic genomes. Their enrichment in satellite repeats and transposable elements (TEs), together with strongly suppressed recombination, has made them difficult to assemble and interpret [4,5]. Meanwhile, these very features make centromeres uniquely informative: repetitive sequences can persist and diverge over long evolutionary timescales, turning centromeric regions into molecular records of chromosome history. This view has been powerfully illustrated in humans. Complete resolution of these regions revealed that human centromeres are not homogeneous blocks of repetitive DNA, but structurally complex and heterogeneous landscapes dominated by α-satellite arrays organized into chromosome-specific higher-order repeats (HORs) [6,7]. These discoveries have transformed centromeres from unresolved gaps in genome assemblies into dynamic genomic domains shaped by repeat amplification, structural variations (SVs), and evolutionary turnover. Additionally, centromeric structures and organisation were also nicely described in plants better related to *Miscanthus* [8]. These advances raise an important question: how have centromeric repeats evolved in polyploid plant genomes with highly diverse genome sizes, repeat landscapes, and chromosome architectures? Deciphering these patterns can therefore provide important insights into ancestry, chromosome differentiation, and genome evolution.

Polyploid genomes offer a unique window into centromere evolution. Polyploidization brings divergent progenitor chromosomes into a single genome, where they are subsequently reshaped through diploidization, repeat turnover, chromosomal rearrangement, and sub-genome differentiation [9,10,11]. For a long time, however, the highly repetitive nature and structural complexity of centromeres made these regions difficult to resolve in polyploid genomes. The integration of long-read sequencing, telomere-to-telomere (T2T) genome assembly, and chromosome-based cytogenetic validation has begun to overcome this barrier, thereby enabling the direct characterization of centromeric repeat organization [3,7]. Recent studies in polyploid plants have revealed centromere dynamics as a powerful lens through which to trace genome evolution after polyploidization. *Saccharum* centromeres show remarkable conservation of satellite repeat distributions despite variation in basic chromosome number and ploidy level, whereas allotetraploid okra and cotton reveal more dynamic patterns, including sub-genome-biased repeat amplification and post-polyploidization centromere remodeling [12,13,14,15]. Together, these findings suggest that centromeres can serve as evolutionary landmarks in polyploid genomes, linking parental origin, sub-genome differentiation, chromosome restructuring, and genome stabilization after polyploidization.

*Miscanthus* (2n = 2*x* = 38), as the grass family, *Poaceae*, belongs to the *Saccharinae* and is closely related to sugarcane and sorghum. It has strong adaptability and stress resistance and shows great potential for bioenergy production [16,17]. Previous studies suggested that its karyotype originated from a sorghum-like ancestor with *n* = 10 and experienced chromosome fusion across *Miscanthus* [16,17,18,19]. Although the parental species contributing the A and B subgenomes have not yet been identified, the subsequent release of reference genomes for *M. sinensis*, *M. lutarioriparius*, and *M. floridulus* further promoted the research of ancient polyploidization and subsequent diploidization on *Miscanthus* [16,17,19]. However, interspecific hybridization and introgression, complex ploidy variation, large genome size, and abundant repetitive sequences continue to hinder high-quality genome assembly in *Miscanthus*, particularly in centromeric and pericentromeric regions. As a result, the evolutionary fate of centromeric repeats during chromosome fusion and subsequent genome restructuring remains largely unresolved. Moreover, centromere organization and evolutionary dynamics in *Miscanthus* remain largely unexplored.

To address these gaps, we combined low-coverage repeatome profiling, genome-wide repeat mapping and cytogenetic validation to investigate centromere-associated satellite repeats in *M. sinensis*. We first used graph-based clustering to identify candidate repetitive sequences and then validated their chromosomal localization by metaphase FISH and fiber-FISH. We further focused on the major centromere-associated satellite repeat MsSat1, characterized its derived variants, examined their subgenome-biased distribution, and analyzed their organization within fusion-derived chromosomal regions. By integrating sequence divergence, subgenome assignment and HOR architecture, we show that MsSat1-derived variants retain signatures of both polyploid subgenome differentiation and chromosome fusion-associated centromere remodeling. In particular, the chromosome 7-specific MsSat1-G variant reveals a distinct satellite-repeat landscape within an expanded centromeric domain of a fusion-derived chromosome. These results provide a cytogenetic and repeatome-based framework for understanding how centromeric satellites are retained, amplified and reorganized during chromosome restructuring in polyploid grasses.

## 2. Results

### 2.1. Graph-Based Clustering Reveals Candidate Centromere-Associated Repeats in Miscanthus

**Figure 1:**
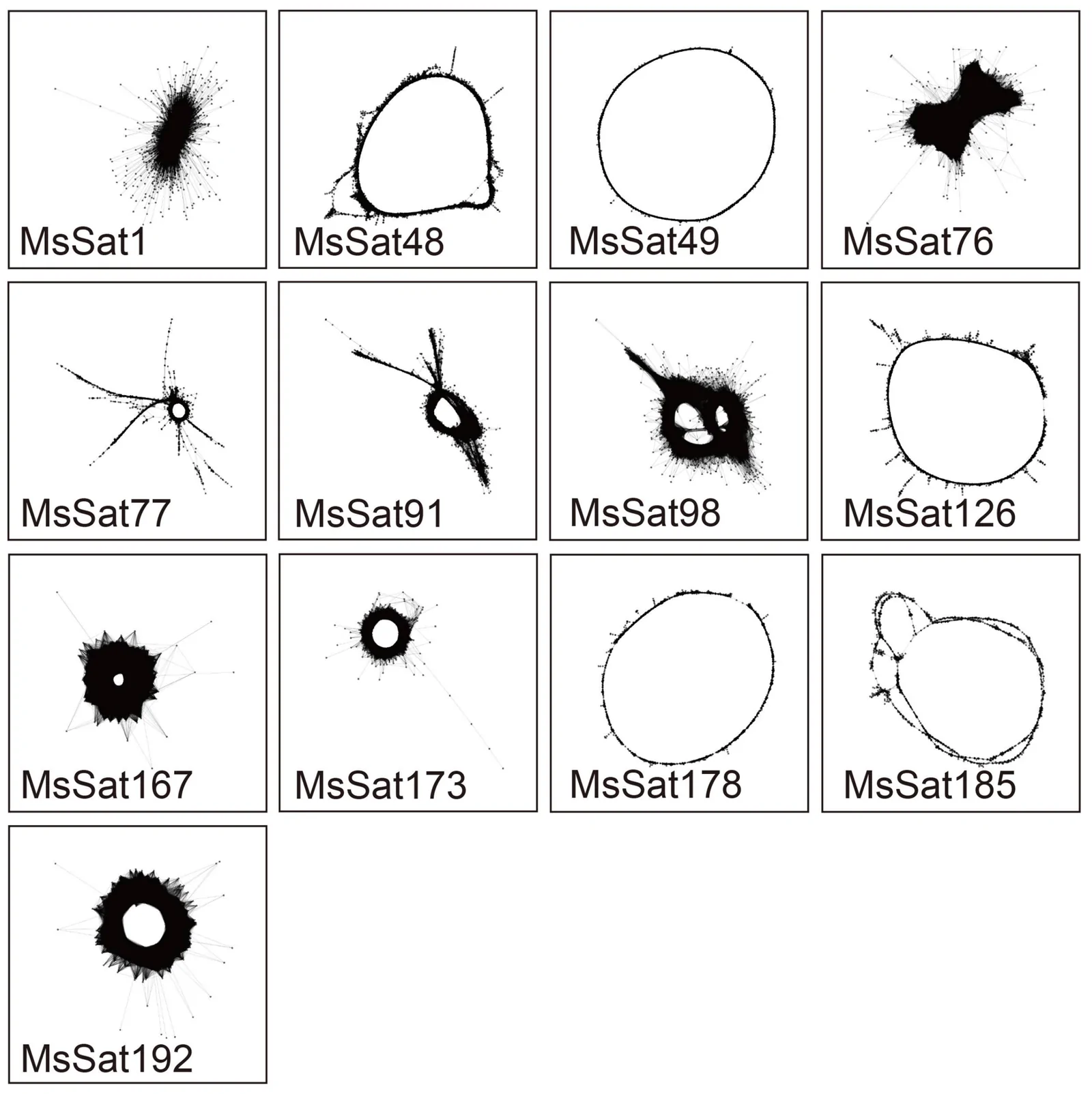
Sequence-similarity network analysis of representative high-copy repeat clusters identified from the *M. sinensis* genome. Each node represents an individual repeat sequence, and edges indicate sequence similarity among repeat units. Thirteen representative repeat clusters (MsSat1, MsSat48, MsSat49, MsSat76, MsSat77, MsSat91, MsSat98, MsSat126, MsSat167, MsSat173, MsSat178, MsSat185, and MsSat192) were selected based on their copy abundance and network organization for subsequent repeat annotation and FISH probe development. Their contrasting network topologies indicate the structural diversity of repetitive sequence families in the *M. sinensis* genome.

To characterize the repetitive DNA landscape of *Miscanthus*, we performed graph-based sequence similarity clustering using published low-coverage Illumina sequencing reads of *M. sinensis*. Two million randomly selected paired-end reads were analyzed with the *RepeatExplorer2* pipeline, and repeat clusters were classified according to graph topology, relative genomic abundance, and sequence features. Clusters showing ring-like or compact dot-like graph topologies were considered putative satellite repeat families, as these structures are typically composed of highly homogeneous tandem repeat sequences [20] (Figure 1). Thirteen representative repeat families were selected for further characterization. Among them, eight repeats were annotated as satellite or satellite-like repeats, including MsSat1, MsSat76, MsSat77, MsSat91, MsSat98, MsSat167, MsSat173 and MsSat192; Four repeats, MsSat48, MsSat126, MsSat178 and MsSat185, were related to retrotransposons, of which MsSat48, MsSat178 and MsSat185 were classified as LTR retrotransposon sequences; Another repeat, MsSat49, was annotated as an rDNA-associated repeat (Table 1).

Interestingly, MsSat1, which accounted for approximately 2.1% of the *M. sinensis* genome, was the most abundant repeat family and contained a 137 bp monomer typical of satellite repeats (Table 1). MsSat76 showed the same monomer size and was identified as an MsSat1-related satellite variant, suggesting diversification within this satellite repeat family. Together, these results revealed a complex repetitive DNA landscape in *M. sinensis*, consisting of satellite repeats, retrotransposon-related sequences and rDNA-associated repeats, providing candidate markers for subsequent cytogenetic localization and centromere-associated repeat analysis.

### 2.2. Cytogenetic Validation of Centromere-Associated Repeats in M. sinensis

**Figure 2:**
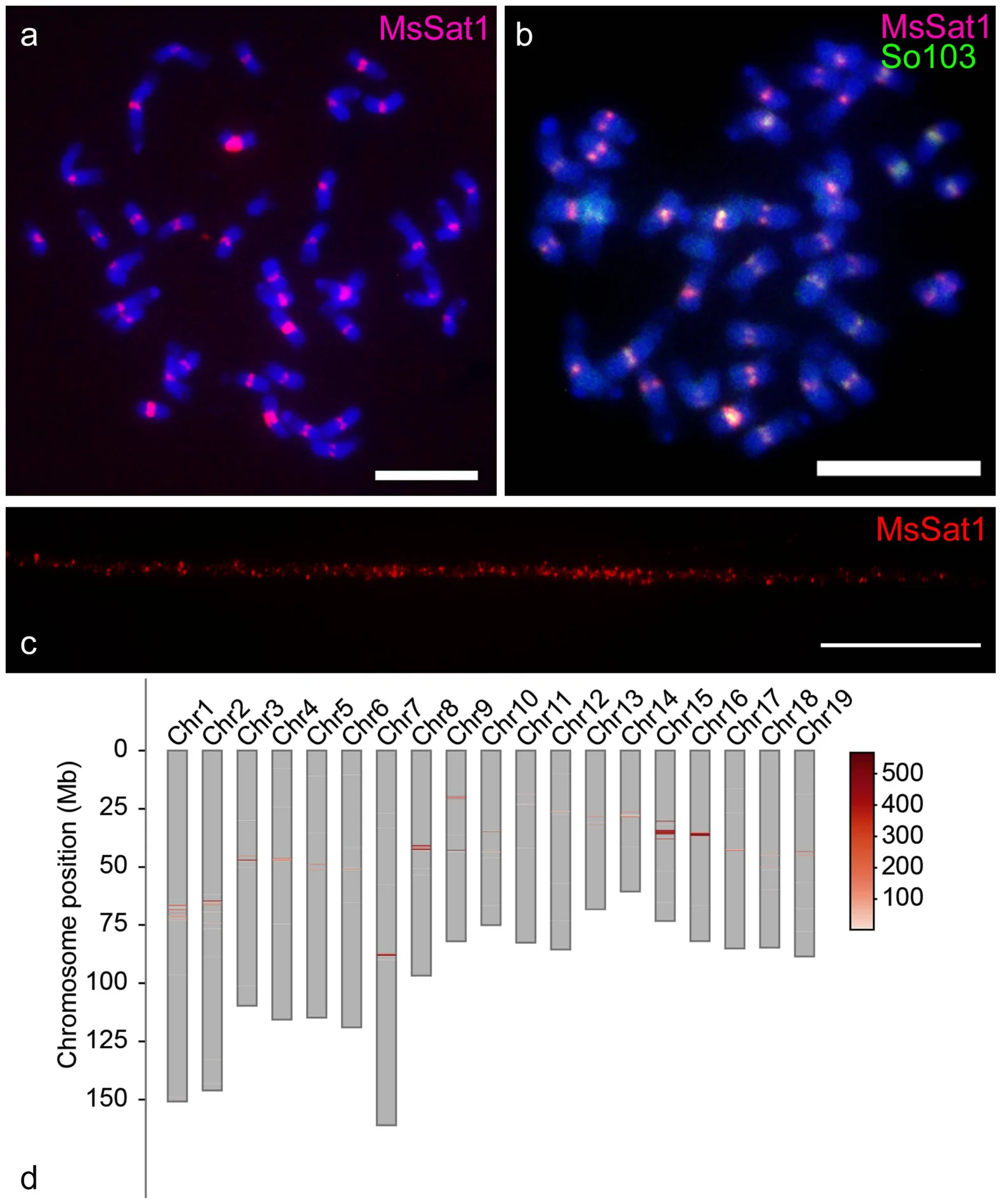
Cytological validation and genomic distribution of MsSat1 in *M. sinensis* accessions of MH142: (**a**) Fluorescence in situ hybridization of the MsSat1 probe on mitotic metaphase chromosomes. MsSat1 signals are shown in magenta, and chromosomes are counterstained with DAPI in blue. (**b**) Dual-color FISH using the MsSat1 and So103 probes on mitotic metaphase chromosomes. (**c**) Fiber-FISH analysis showing that MsSat1 forms long and continuous tandem arrays. (**d**) Genome-wide distribution of MsSat1-related sequences across chromosomes Chr1–Chr19. Signal intensity indicates the relative enrichment of MsSat1 sequences along each chromosome, with strong accumulation around centromeric or pericentromeric regions.

As cytological localization provides the most direct evidence for assessing whether repetitive sequences are physically associated with centromeric regions, we performed fluorescence in situ hybridization (FISH) using 13 representative candidates repeat probes, including MsSat1, MsSat48, MsSat49, MsSat76, MsSat77, MsSat91, MsSat98, MsSat126, MsSat167, MsSat173, MsSat178, MsSat185 and MsSat192 (Table S1 and Figure S2). FISH signals produced a specific distinct signal on each chromosome, suggesting that MsSat1 represents a major centromere-associated satellite repeat in *M. sinensis*, consistent with the results of in silico mapping analysis (Figure 2a,d). Grasses are known to harbour centromeric retrotransposon (CR) in their centromeric and pericentric regions. Furthermore, the signals of the *Saccharum officinarum* So103 probe [14] nearly completely overlapped with the MsSat1 FISH signals, supporting the localization of MsSat1 within centromeric chromatin and indicating that MsSat1 and CR elements occupy closely associated centromeric domains in *M. sinensis* (Figure 2b). In addition, fiber-FISH analysis showed that MsSat1 formed continuous signals on DNA fibers, indicating that MsSat1 is arranged in tandem repeats and further confirming that MsSat1 is a major centromeric satellite repeat in *M. sinensis* (Figure 2c).

Although MsSat76 showed a FISH signal pattern similar to that of MsSat1, its genomic proportion was only 0.32%, much lower than that of MsSat1 (2.1%), probably as a result that MsSat76 as a low-abundance variant to the MsSat1-related centromeric satellite repeat family in *Miscanthus* (Figure S1 and Table 1). In contrast, the remaining repeat sequences showed non-centromeric distribution patterns, with FISH signals either dispersed along chromosome arms or concentrated at telomeric or sub-telomeric regions (Figure S2). Additionally, MsSat49 and MsSat77, which were annotated as rDNA-associated repeats, each produced two discrete FISH signals on metaphase chromosomes, consistent with the signal patterns reported in previous studies [18] (Figure S3 and Table 1).

### 2.3. MsSat1-Based Clustering Identifies Centromere-Associated Variants in M. sinensis

To identify MsSat1-related repeat variants enriched in centromeric regions, we performed de novo similarity-based clustering using MsSat1-derived sequences and then evaluated their enrichment across the *M. sinensis* genome. A total of 93,020 repeat clusters were generated, and representative sequences from the top 13 most abundant clusters, with homology greater than 85%, were selected as putative MsSat1 variant sequences for further analysis (Figure S4, Tables S2 and S3). Genomic mapping also demonstrated clustered alignments in the centromeric regions for all of these repeats (Figure S5). FISH results validated these genome alignments, with 13 repeats producing from 2 to 38 centromeric signals in *M. sinensis* metaphase chromosomes, respectively (Figure S6).

**Figure 3:**
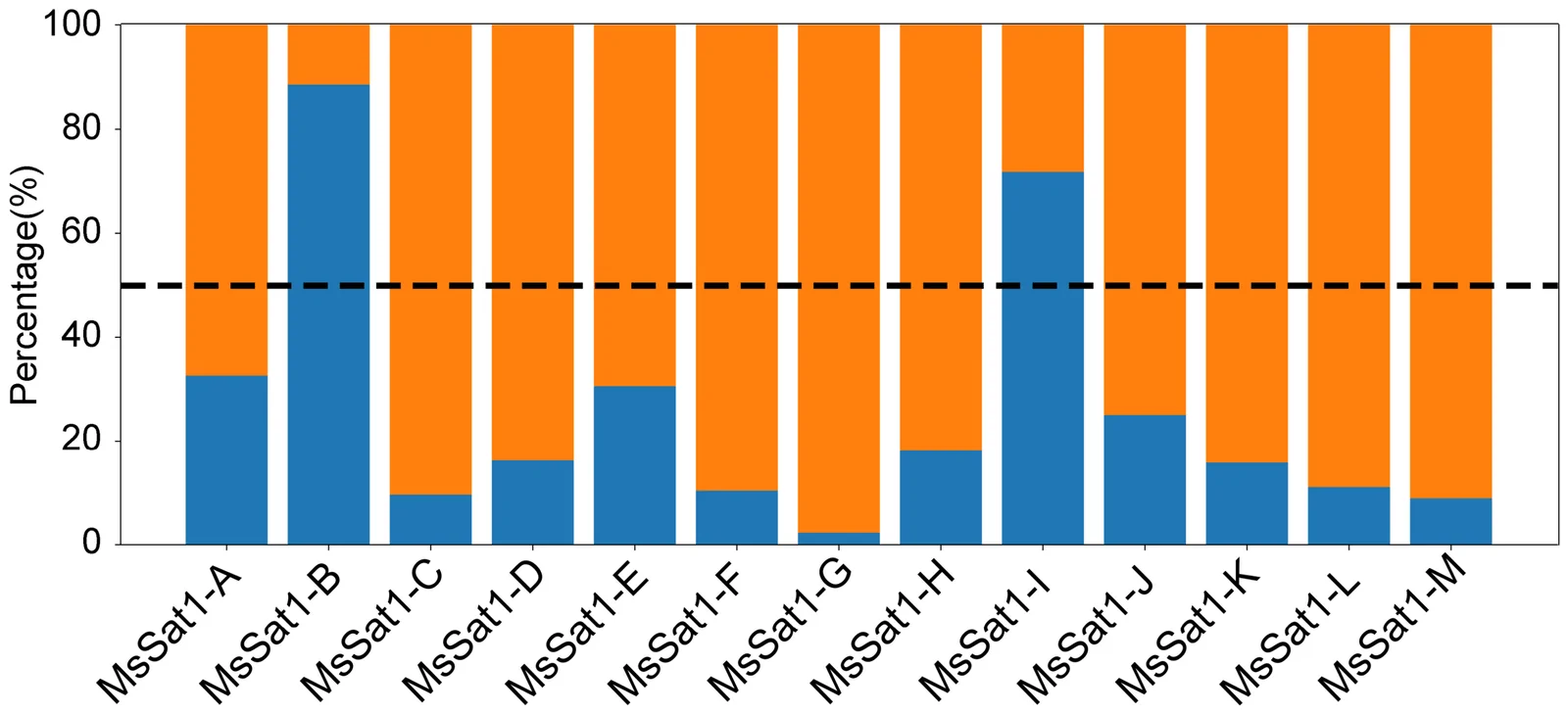
Subgenome-biased distribution of MsSat1-derived variants MsSat1A–M. Stacked bar plots showing the relative proportions of MsSat1-derived variants assigned to the two subgenomes of *M. sinensis*. The dashed line indicates the expected 50% distribution under no subgenome bias. Orange color indicates subgenome B, and blue color indicates subgenome A.

To further examine whether these MsSat1-derived variants showed subgenome-biased distribution, we assigned their genomic alignments to the A and B subgenomes. As the blast result shown that the distribution of these variants was highly asymmetric: most variants were preferentially enriched in the B subgenome, including MsSat1-C, MsSat1-D, MsSat1-F, MsSat1-G, MsSat1-H, MsSat1-K, MsSat1-L and MsSat1-M, whereas only Ms1-B and Ms1-I showed a clear A-subgenome-biased distribution (Figure 3). Notably, MsSat1-G exhibited the strongest B-subgenome bias, with 97.57% of its alignments assigned to the B subgenome, consistent with the alignment result (Figure 3 and Figure S5).

### 2.4. Fusion-Derived Chromosomes Harbor Distinct MsSat1-G Variants and Diversified HOR Architectures

**Figure 4:**
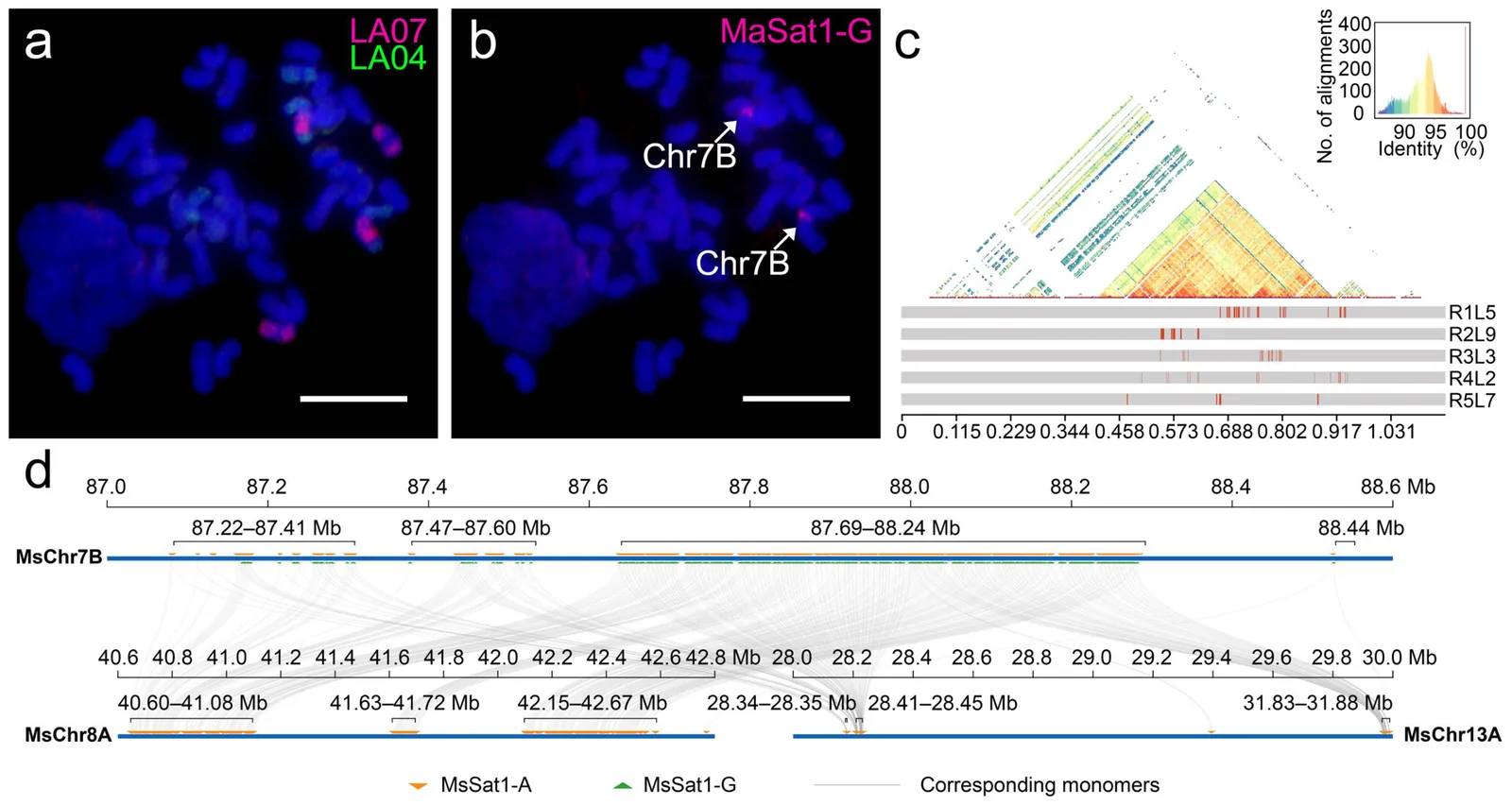
Chr7-specific MsSat1-G satellite variant associated with chromosome fusion: (**a**) *S. officinarum*-specific oligo probes painting on mitotic metaphase chromosomes of MH142. (**b**) FISH of the MsSat1-G probe on mitotic metaphase chromosomes of *M. sinensis* accessions of MH142.white arrow highlights the *M. sinensis* of chromosome 7B. MsSat1-G signals are shown in red, and chromosomes are counterstained with DAPI in blue. (**c**) Sequence-similarity dot plot and monomer alignment of the MsSat1-G array on the fusion chromosome Chr7, showing its HOR organization. The inset histogram shows the sequence identity distribution of aligned monomers. Both axes show genomic positions along the same array. Colored points represent local pairwise alignments, with different colors indicating different levels of sequence identity, as shown in the inset histogram. The inset shows the distribution of alignment identities. Recurrent diagonal and off-diagonal blocks reveal the internal organization of canonical and nested HOR structures. (**d**) Genomic localization and comparative mapping of MsSat1 and MsSat1-G monomers across Chr07, Chr08 and Chr13. Orange and green markers indicate MsSat1 and MsSat1-G monomers, respectively, and grey lines connect corresponding monomers between chromosomal regions. The Chr07-enriched MsSat1-G array indicates local satellite remodeling associated with the formation and evolution of the fusion-derived Chr7 centromeric region. Scale bars = 10 μm in (**a**,**b**).

Among the MsSat1-derived variants, MsSat1-G displayed the most striking chromosome-specific distribution. Genome-wide mapping showed that MsSat1-G aligned exclusively to chromosome 7, which was derived from a fusion between ancestral chromosomes A and B (Figure S5). Consistently, FISH analysis detected a chromosome-specific signal corresponding to this chromosome (Figure 4a,b), indicating that MsSat1-G represents a rare MsSat1 variant associated with the centromeric region of the fusion-derived chromosome 7.

Further detailed analysis of the chromosome 7 centromeric region revealed that Ms1-G was not present as an isolated repeat, but was organized together with MsSat1-related sequences in parallel tandem arrays, indicating that this chromosome-specific variant was incorporated into an expanded MsSat1-rich repeat domain after chromosome fusion (Figure 4d). On chromosome 7, these repeats occupied three major blocks at 87.22–87.41 Mb, 87.47–87.60 Mb and 87.69–88.24 Mb, together covering approximately 0.87 Mb within a ~1.0 Mb centromeric repeat-rich domain (Figure 4d). In contrast, the corresponding regions on the pre-fusion chromosomes were more fragmented and less continuous. The expanded repeat domain on chromosome 7 indicated that chromosome fusion was accompanied by local amplification and reorganization of MsSat1-derived centromeric repeats.

**Figure 5:**
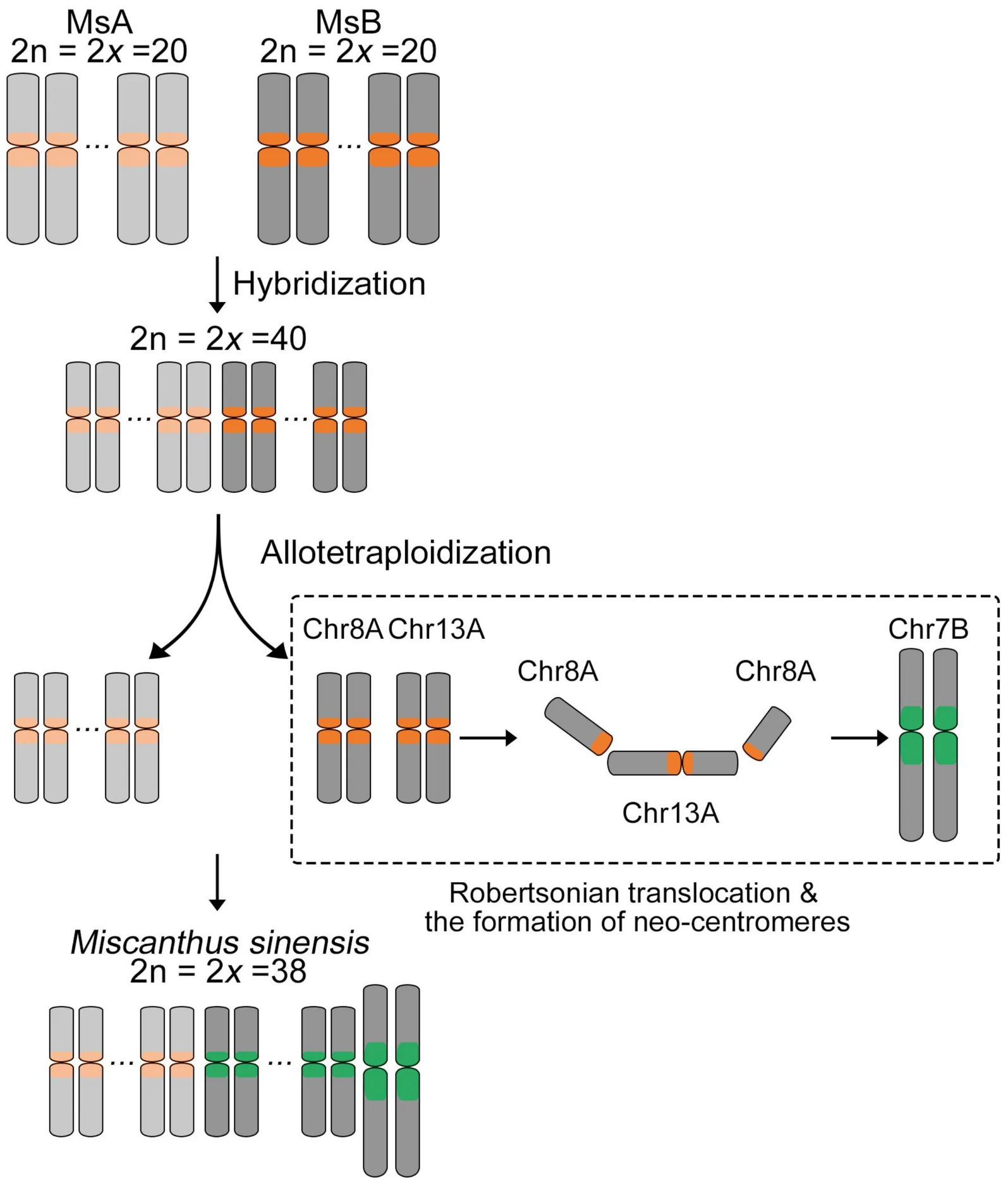
A proposed model for asymmetric centromeric satellite evolution after allopolyploidization. Following allotetraploidization, chromosome rearrangements [19], including Robertsonian translocation, promoted local centromere remodeling. Such remodeling may have involved the inactivation of ancestral centromeres and the formation of neo-centromeres marked by newly amplified satellite variants, ultimately shaping the subgenome-biased centromeric repeat landscape observed in *M. sinensis*.

HOR organization provides a sensitive readout of centromeric satellite evolution, capturing how repeat monomers are amplified, homogenized and reorganized within satellite arrays during chromosome diversification. To determine whether MsSat1 and MsSat1-G differ in their higher-order architecture, we independently annotated HOR structures for the two satellite arrays (Figure 4c). In MsSat1, 319 HOR records were identified, with a pronounced enrichment of R2L2, which accounted for 36.99% of all HORs, followed by R1L5 at 21.32% (Figure S7). The four most abundant HOR classes collectively represented 72.73% of the Ms1 HOR repertoire, indicating a relatively constrained and regular higher-order organization (Figure S7). By contrast, MsSat1-G contained 261 HOR records and displayed a more heterogeneous HOR composition. Its most abundant class, R1L5, accounted for only 20.69%, followed by R4L2, R3L3 and R6L2, with the top four classes together representing 59.77% of all MsSat1-G HORs (Figure 4d and Figure S8). Although dimeric remained the most frequent length class in MsSat1-G, their proportion was reduced to 30.65%, whereas pentameric and trimeric contributed 24.90% and 13.79%, respectively. These results indicate that MsSat1-G preserves a short HOR-based organization but has a reduced dominance of dimeric HORs and a broader spectrum of HOR architectures, implying that chromosomal fusion may create a distinct evolutionary context for centromeric satellite turnover, in which both satellite sequence variants and their HOR array are remodeled during the diversification and stabilization of fusion-derived chromosomes (Figure 5).

### 2.5. The Evolution of Centromere Satellite Repeats Across M. sinensis

**Figure 6:**
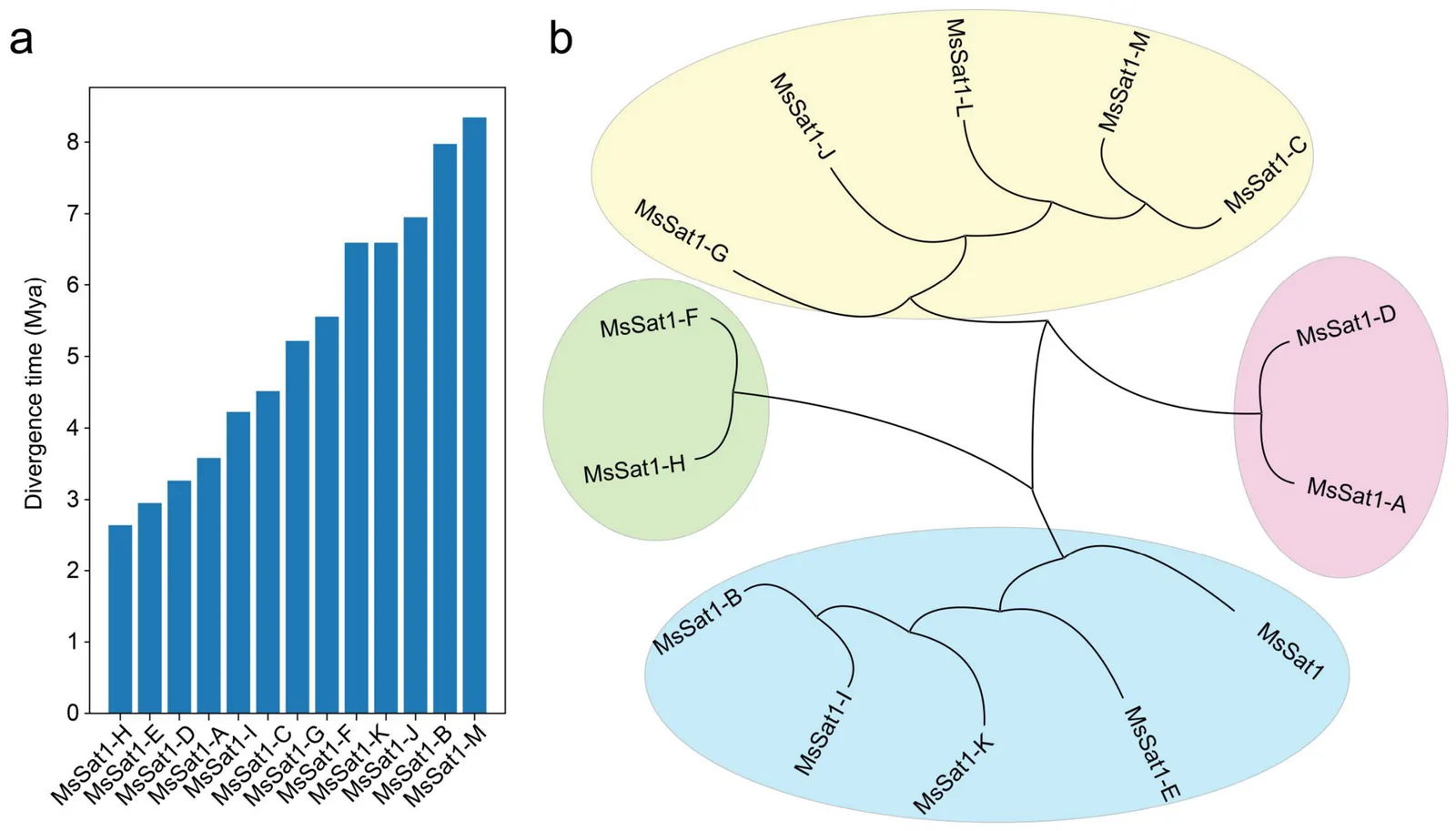
Evolutionary divergence and phylogenetic relationships of MsSat1-derived variants. (**a**) Estimated divergence times of MsSat1-derived variants, showing that different variants originated at distinct evolutionary stages. (**b**) Phylogenetic tree constructed from MsSat1-derived variant sequences. The variants are grouped into several major clades, indicating that the present MsSat1 repeat family contains multiple evolutionarily differentiated lineages.

To explore the evolutionary relationships among MsSat1-derived centromeric satellite variants, we estimated their divergence times and constructed a phylogenetic tree based on representative variant sequences. The estimated divergence times of MsSat1-derived variants ranged from approximately 2.6 to 8.3 MYA, revealing substantial temporal heterogeneity among these satellite variants. Ms1-G diverged at approximately 5.2 MYA, placing it in an intermediate position within the MsSat1 variant repertoire (Figure 6a). Phylogenetic analysis further separated these variants into several distinct groups, including closely related pairs such as MsSat1-A/MsSat1-D and MsSat1-F/MsSat1-H, as well as larger clades containing multiple MsSat1-related variants (Figure 6b). Notably, Ms1-G occupied a distinct phylogenetic position within the MsSat1-derived variants and also showed a chromosome 7-specific distribution, distinguishing it from the broadly distributed MsSat1-related variants. These features suggest that MsSat1-G represents a distinct MsSat1-derived lineage associated with the fusion-derived chromosome 7.

## 3. Discussion

Centromere-specific Histone H3 chromatin immunoprecipitation (CENH3-ChIP) offers powerful strategies for centromere study [21,22]. In practice, however, ChIP-based approaches require robust CENH3 antibodies and optimized chromatin assays, and depend on nearly complete genome assemblies [22,23]. These requirements remain difficult to meet in many non-model plants, especially in crops lacking high-quality reference genomes and in polyploid species with large, repeat-rich genomes. In this study, we adopted a complementary strategy based on low-coverage whole-genome Illumina reads and graph-based sequence similarity clustering (see Section 4.2). Typically requiring only approximately 0.3× genome coverage, this approach does not rely on prior centromere annotation, species-specific CENH3 antibodies or complete centromere assemblies. Instead, it takes advantage of the high abundance and sequence homogeneity of satellite DNA to recover candidate centromeric repeat families directly from unassembled reads (Figure 1 and Table 1).

Using this strategy, we identified MsSat1 as the most abundant satellite repeat in *Miscanthus* and confirmed its centromeric localization by metaphase FISH and fiber-FISH, with its close overlap with the centromeric retrotransposon probe further supporting its association with centromeric domains (Figure 2). Importantly, this workflow also allowed us to recover low-abundance MsSat1-related variants, including a Chr7-specific MsSat-G variant, which may have been missed or underestimated by assembly-based analyses alone. Thus, low-coverage repeatome profiling, when coupled with cytogenetic validation, provides a practical and scalable route for identifying candidate centromere-associated repeats in complex polyploid genomes [24]. Nevertheless, this strategy identifies candidate centromere-associated repeats rather than directly defining functional centromeric DNA. Therefore, cytological localization and, where possible, chromatin-based validation remain important for distinguishing true centromere-associated repeats from other abundant repetitive sequences. Although our analyses resolve the chromosome-scale distribution and fusion-associated remodeling of centromeric repeats, they are still not accurate enough. Future integration of these approaches with T2T genome assemblies, long-read sequencing, CENH3 ChIP-seq, and high-resolution comparative analyses will be essential for resolving centromere size, sequence composition, and functional organization in *M. sinensis*.

The chromosome 7-specific MsSat1-G variant was embedded within MsSat1 tandem arrays in an expanded centromeric repeat domain rather than forming a separate repeat block (Figure 4a,b,d). Together with its evolutionary divergence from the major MsSat1 lineage (Figure 6), this pattern argues against a simple insertion or transposition event from an ancestral chromosome and instead supports a model in which MsSat1-G was derived from a pre-existing MsSat1-related lineage that was retained and amplified during post-fusion centromere remodeling. This remodeling was also reflected in HORs: the MsSat1-G-associated region showed a more heterogeneous HOR composition than the major MsSat1 array, suggesting that chromosome fusion reshaped not only satellite abundance and distribution, but also the internal architecture of centromeric repeats (Figure 4b). Notably, MsSat1-derived HORs in *Miscanthus* were dominated by dimeric and pentameric units, resembling the organizational logic of human α-satellite HORs [6,7], differs from the monomeric expansion reported in recent plant examples such as *Arabidopsis* and cotton [15,25]. This finding suggests that plant centromeric satellites may evolve through more diverse HOR organizational routes than previously appreciated. Whether different HOR architectures influence centromere activity, chromatin organization, or long-term chromosome stability remains an important question for future studies. The current HOR profiles are based on a non-T2T assembly and may therefore underestimate their full complexity, which future T2T assemblies will help resolve in *M. sinensis*.

Stable chromosome fusion requires resolution of a fundamental centromere problem: two ancestral chromosomes initially contribute two centromeres to a single fusion chromosome, whereas only one functional centromere is generally maintained [26]. This principle is exemplified by human chromosome 2, in which one ancestral centromere remains functional while the other has degenerated [27,28]. A similar centromere-resolution process has recently been revealed in the *Saccharum complex*. In *Erianthus rockii* and *Narenga porphyrocoma*, fusion-derived chromosomes retain only one active centromere, whereas the redundant ancestral centromere becomes inactivated and loses its associated centromeric repeats [13,18]. Consistent with this model, our cytological observations in *M. sinensis* did not reveal dicentric chromosome structures, indicating that chromosome fusion was accompanied by centromere resolution rather than stable maintenance of two centromeres. Our results extend this view by showing that the fusion-derived chromosome 7 harbors the chromosome-specific MsSat1-G variant within an expanded MsSat1-rich domain, suggesting that the retained centromeric region underwent local satellite-repeat remodeling and acquired a distinct centromeric satellite signature. Thus, centromere inactivation, repeat loss, and repeat amplification may represent different but related outcomes of fusion-associated centromere evolution in polyploid grasses.

The subgenome-biased distribution of MsSat1-derived variants indicates that centromeric satellite repeats evolved asymmetrically after polyploidization in *M. sinensis*, suggesting that the A and B subgenomes experienced distinct trajectories of centromeric repeat evolution (Figure 3). Comparable patterns have been observed in other polyploid plants, such as wheat, teff, cotton and okra, in which centromeric satellites or retrotransposons display subgenome-specific accumulation, turnover or differentiation [12,15,29,30]. These examples suggest that centromeric repeats are highly dynamic after polyploid formation and can preserve molecular signatures of subgenome divergence. Together with its chromosome 7-specific localization within an expanded MsSat1-rich domain, the strong B-subgenome enrichment of MsSat1-G further suggests that subgenome-biased satellite amplification may have been coupled with fusion-associated centromere remodeling. Such biased repeat remodeling may be relevant to genome stability, because centromeric satellite arrays are closely linked to centromere identity, kinetochore assembly and faithful chromosome segregation, particularly in fusion-derived chromosomes where redundant ancestral centromeres must be resolved [31,32]. However, whether this B-subgenome bias reflects ancestral repeat divergence, post-polyploidization amplification, preferential repeat loss from the A subgenome, or local expansion after chromosome fusion remains unresolved. It is also unclear whether MsSat1-G directly contributed to stabilization of the retained centromere or instead represents a consequence of centromere remodeling after fusion. Thus, MsSat1-derived variants provide a repeat-level signature of subgenome differentiation and chromosome restructuring, while also highlighting the need to distinguish inherited centromeric repeat variation from newly remodeled repeat landscapes in fusion-derived chromosomes.

## 4. Materials and Methods

### 4.1. Plant Materials

The *M. sinensis* accessions M142 (Figure S9) used for FISH analysis were grown in the greenhouse of the College of Bioengineering (Hengyang, Hunan Province, China). Actively growing root tips were collected from these plants and used for metaphase chromosome preparation.

### 4.2. Identification of Repetitive Sequences

To identify potential centromeric repeat sequences in the M142 genome, the RepeatExplore2 platform (ftps://repeatexplorer-elixir.cerit-sc.cz, accessed on 15 January 2026) was employed to detect and classify repetitive elements. A random subset of 4,000,000 sequencing reads (SRR10829158) was analyzed through the RepeatExplorer2 online pipeline to generate genome-wide repeat clusters. Tandem repeat types within each cluster were classified using the TAREAN module. To further investigate the diversity of the centromeric satellite sequence MsSat1, 40,000,000 paired-end 150 bp sequencing reads from M142 high-throughput sequencing data were randomly selected and aligned to the Ms1 sequence using LASTZ (v1.04.52) [33]. All Ms1 homologous sequences contained fewer than 10 gaps and ranged from 125 to 145 bp in length. The sequences were subjected to similarity-based clustering analysis, and after two iterative rounds, 13 distinct repeat variant clusters were identified and designated as MsSat1-A to MsSat1-M. Monomer sequences of each variant cluster were aligned using DNAMAN software to analyze sequence conservation and variability.

### 4.3. Probe Preparation

The MsSat1-MsSat192 sequences were PCR-amplified from M142 genomic DNA, purified, and used for probe labeling. In contrast, the MsSat1-A to MsSat1-M sequences were chemically synthesized. All sequences were labeled with DIG-11-dUTP (Roche Diagnostics, Basel Switzerland, https://www.roche.com/about/business/diagnostics.htm, accessed on 15 January 2026) via the nick translation method. Oligo library and probe preparation of the specific *S. officinarum* oligo LA04 and LA07 probe were referred to published protocols. LA07 probe was labled with Cy3-dUTP (red) and LA04 probe was labled with FITC-dUTP (green).

### 4.4. Chromosome Preparation and FISH

Root tips were pretreated with 8-hydroxyquinoline at room temperature for 2 h and then fixed in Carnoy’s solution (ethanol:glacial acetic acid, 3:1) for 24 h. The fixed root tips were digested with an enzyme mixture containing 1% pectolyase Y-23, 2% pectolyase, 2% cellulase RS, and 4% Onozuka R-10 cellulase at 37 °C for 3 h. The resulting cell suspension was dropped onto glass slides. The fiber chromosome was performed following previously established protocols (ref.). FISH was performed following previously established protocols [13,15,34]. Denatured probes (100 ng of probe) were combined with a hybridization buffer containing 50% formamide and 20% dextran sulfate in 2 × SSC and applied to denatured chromosomal spreads. Slides were covered with coverslips and incubated overnight at 37 °C in a humid chamber. Post-hybridization washes were performed three times in 2 × SSC for 5 min each, followed by a single 5 min wash in 1 × PBS at room temperature. Hybridization signals were detected using rhodamine-conjugated anti-digoxigenin antibodies (Roche Diagnostics) or Alexa Fluor™ 488 streptavidin (Life Technologies, now Thermo Fisher Scientific, Waltham, MA, USA) for biotin-labeled probes. Chromosomes were counterstained with DAPI in Vectashield antifade solution. Images were captured using an Olympus BX63 fluorescence microscope (Olympus Corporation, Tokyo, Japan) equipped with a DP80 CCD camera and processed with cellSens Dimension 1.9, with contrast adjustments made in Photoshop CC to enhance clarity.

### 4.5. Genome-Wide Mapping and Subgenome Assignment of Ms1-Derived Variants

Representative monomer sequences of Ms1-derived variants were used as queries for genome-wide alignment against the *M. sinensis* reference genome using BLASTN software [35]. High-confidence alignments with appropriate monomer length and limited gaps were retained and converted into BED format for chromosomal distribution analysis. To evaluate subgenome-biased distribution, retained alignments were assigned to the A and B subgenomes according to the published chromosome classification of *M. sinensis*. The number and proportion of alignments for each variant were then calculated separately for the two subgenomes.

### 4.6. HOR Analysis, Divergence Time Estimation and Phylogenetic Analysis

Genomic regions enriched for Ms1 and Ms1-G were extracted based on genome-wide alignment coordinates and used for HOR analysis. HORs were identified using HiCAT *v.1.1.0* [25], and tandem repeat structures were visualized using StainedGlass *v.0.5* [36] with a window size of 2000 bp. The abundance and composition of HOR classes were compared between Ms1 and Ms1-G arrays. For evolutionary analysis, representative sequences of Ms1-derived variants were aligned using DNAMAN software. Pairwise sequence divergence was calculated using the Kimura two-parameter model, and approximate divergence times were estimated using the formula **T = *K*/*2r***, where ***K*** is corrected sequence divergence and ***r*** is the neutral substitution rate. A neighbor-joining phylogenetic tree was constructed based on the aligned variant sequences with 1000 bootstrap replicates and visualized using iTOL.

## Figures and Tables

**Table 1:** Composition and density of clustered repeat sequences based on NGS-seq reads in *Miscanthus*.

Repeat	Genom Proportion (%)	GC Content (%)	Monomer Size (bp)	Repeat Type
MsSat1	2.1	50	137	satellite repeat
MsSat48	0.38	61	4746	LTR retrotransposon
MsSat49 (5S rDNA)	0.38	58	7657	rDNA-associated repeat
MsSat76	0.32	52	137	Ms1-related satellite variant
MsSat77 (35S rDNA)	0.32	32	677	rDNA-associated repeat
MsSat91	0.28	66	818	satellite repeat
MsSat98	0.27	38	440	satellite repeat
MsSat126	0.18	64	5328	retrotransposon
MsSat167	0.09	48	185	satellite repeat
MsSat173	0.075	48	368	satellite repeat
MsSat178	0.068	44	6587	LTR retrotransposon
MsSat185	0.062	43	4446	LTR retrotransposon
MsSat192	0.053	57	300	satellite repeat

## Data Availability

The reference genome of *M. sinensis* DH1 is available at NCBI under accession PRJNA346689.

## Supplementary Materials

The following supporting information can be downloaded at: https://www.mdpi.com/article/10.3390/plants15182855/s1, Figure S1. FISH validation of MsSat76 to *M. sinensis* accessions of MH149.Chromosomes were counterstained with DAPI, Bar = 10 μm. Figure S2. FISH screening of selected satellite repeats to *M. sinensis* accessions of MH149. Representative FISH patterns of additional MsSat repeat candidates on metaphase chromosomes. Probe names are indicated in each panel. Chromosomes were counterstained with DAPI, Bar = 10 μm. Figure S3. Chromosomal localization of 5S rDNA (MsSat49) and 35S rDNA (MsSat77) to *M. sinensis* accessions of MH149. FISH mapping of 5S rDNA and 35S rDNA loci on metaphase chromosomes (a). 5S rDNA and 35S rDNA signals are shown in red and green (b), respectively. Chromosomes were counterstained with DAPI, Bar = 10 μm. Figure S4. Sequence alignment of MsSat1-related repeats. Alignment of representative MsSat1-related sequences showing the conserved region used for probe design. Figure S5. Genome-wide distribution of MsSat1-derived variants. Chromosomal distribution of MsSat1-derived variants across the *M. sinensis* genome. Different colors indicate different variant types. Figure S6. FISH validation of MsSat1-derived variants to *M. sinensis* accessions of MH149. Representative FISH signals of MsSat1-derived variants on metaphase chromosomes. The number of detected signals is indicated in each panel. Chromosomes were counterstained with DAPI, Bar = 10 μm. Figure S7. HOR organization of MsSat1 arrays. (a) Numbers of canonical and nested repeats in different HOR types. R indicates the repeat-pattern type and L indicates the number of monomers comprising the corresponding HOR unit. (b) Dot-plot visualization showing the organization of representative MsSat1 HOR arrays. Both axes show genomic positions along the same array. Colored points represent local pairwise alignments, with colors indicating sequence identity. The inset shows the distribution of alignment identities. Recurrent diagonal and off-diagonal blocks reveal the internal organization of canonical and nested HOR structures. Figure S8. Quantification of canonical and nested HOR repeats. R1L5, R2L2, R3L9, R4L3, and R6L7 denote representative MsSat1 HOR arrays, where R indicates the repeat-pattern type and L indicates the number of monomers comprising the corresponding HOR unit. Bar plot showing the repeat numbers of canonical and nested structures among different HOR types. Figure S9. Phenotypes of M. sinensis accessions M142. Bar = 10 cm. Table S1. The information of representative candidate satellite sequence from first cluster. Table S2. The information of representative candidate variation satellite sequence from second cluster. Table S3. The PCR primers used for development of FISH probes for representative candidate variation satellite sequence.
