# Supplementary material for: Fusion-Derived Centromeres Reveal Satellite Repeat Remodeling in Miscanthus sinensis
Source: Plants (Basel). 2026 Sep 18;15(18):2855. doi: 10.3390/plants15182855 (PMC13610342; doi:10.3390/plants15182855)
Supplement: Supplementary file 1 [file plants-15-02855-s001.zip › Supplementary Figure.pdf]

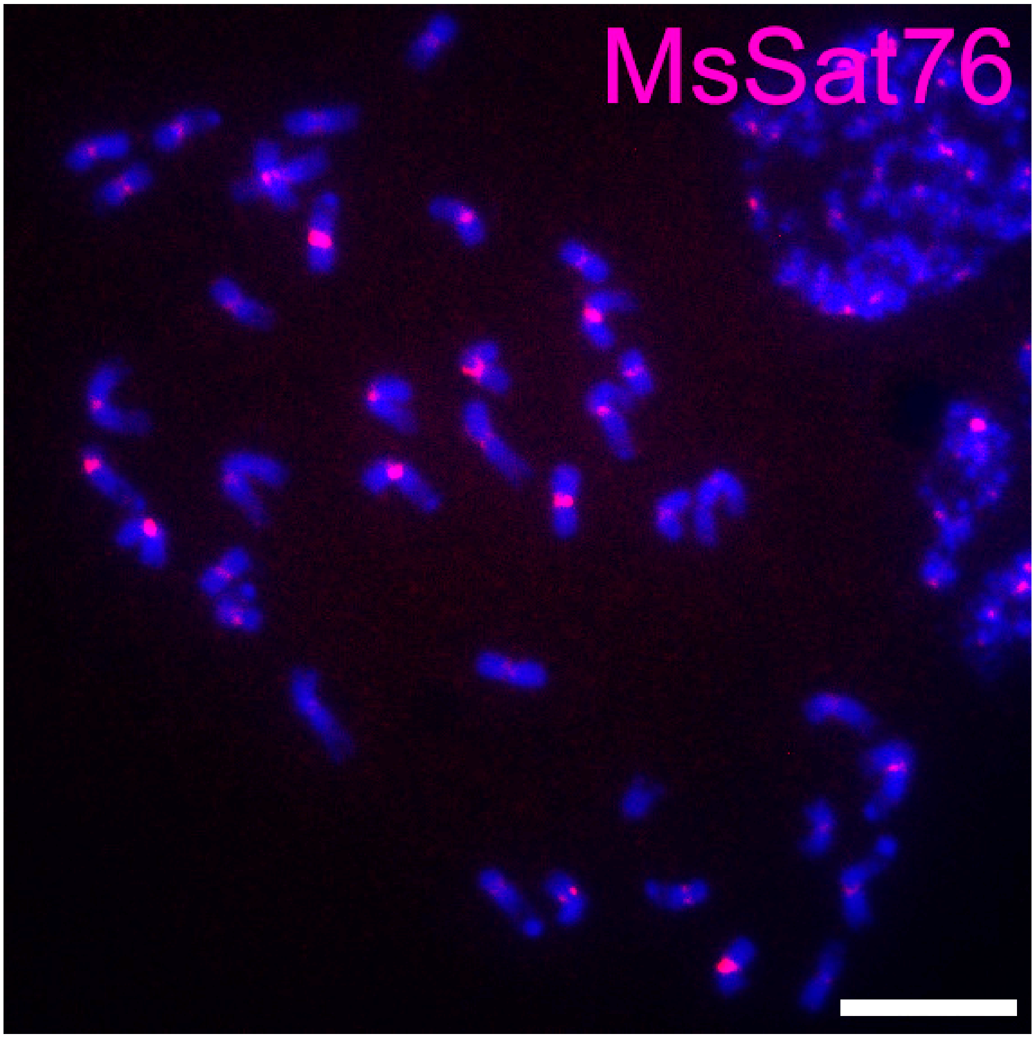

Figure S1. FISH validation of MsSat76 to *M. sinensis* accessions of MH149.Chromosomes were counterstained with DAPI, Bar = 10μm.

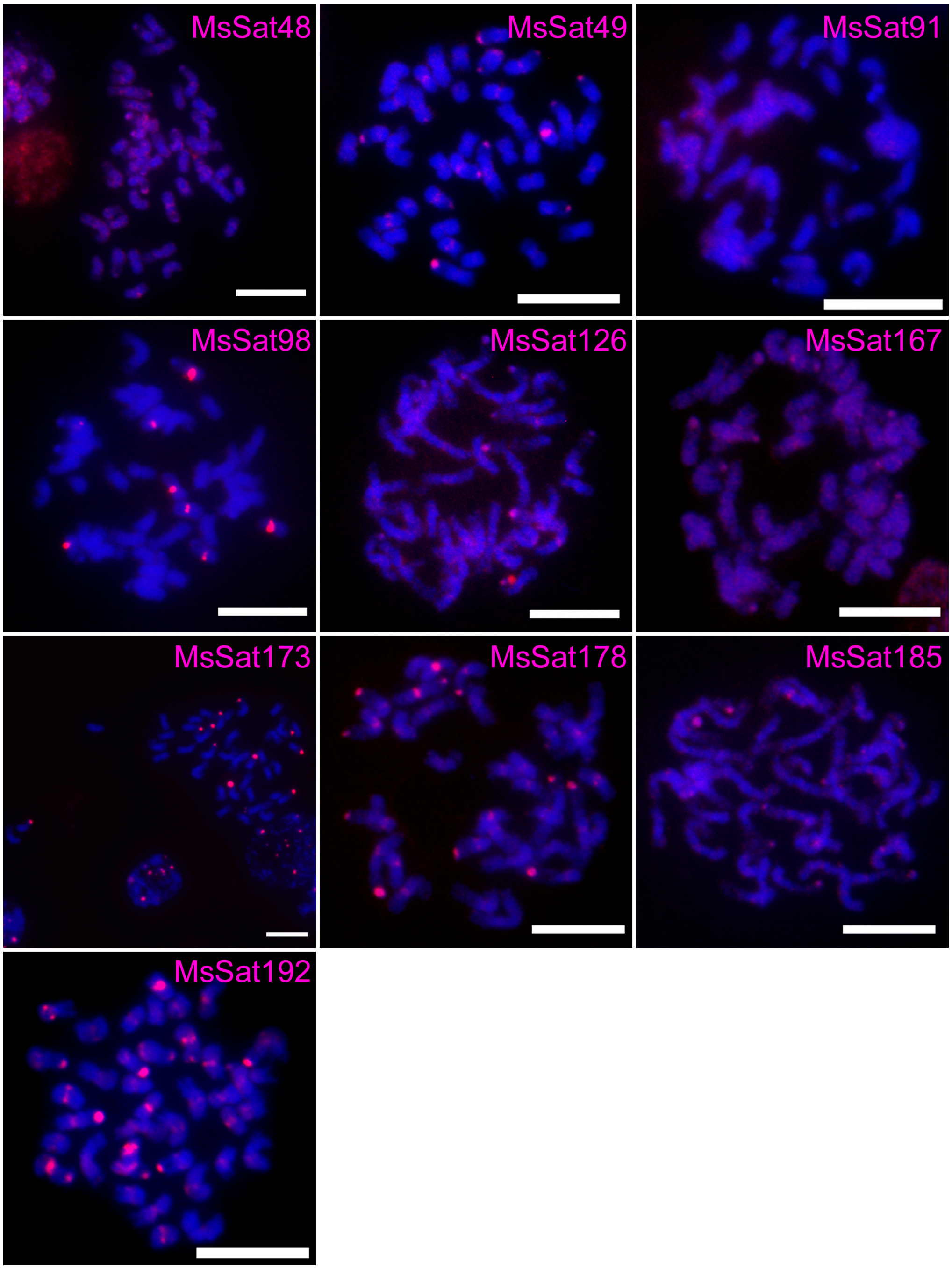

Figure S2. FISH screening of selected satellite repeats to *M. sinensis* accessions of MH149. Representative FISH patterns of additional MsSat repeat candidates on metaphase chromosomes. Probe names are indicated in each panel. Chromosomes were counterstained with DAPI, Bar = 10μm.

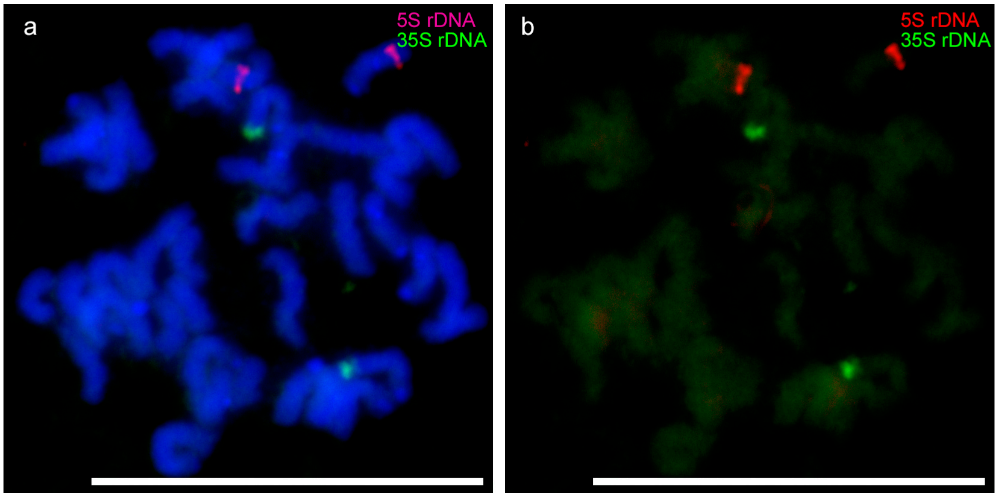

Figure S3. Chromosomal localization of 5S rDNA (MsSat49) and 35S rDNA (MsSat77) to *M. sinensis* accessions of MH149. FISH mapping of 5S rDNA and 35S rDNA loci on metaphase chromosomes (a). 5S rDNA and 35S rDNA signals are shown in red and green (b), respectively. Chromosomes were counterstained with DAPI, Bar = 10μm.

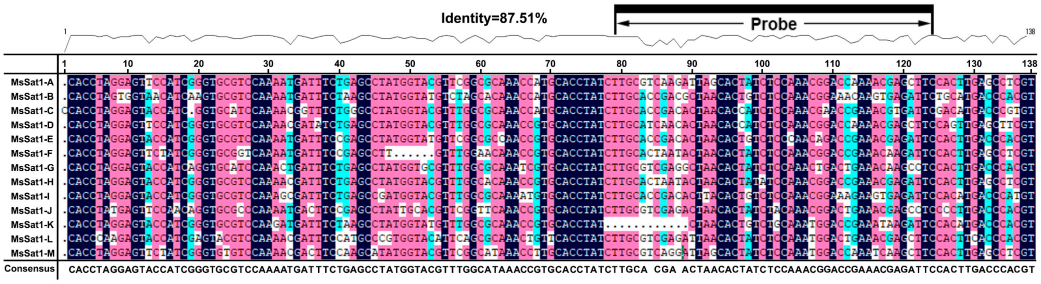

Figure S4. Sequence alignment of MsSat1-related repeats. Alignment of representative MsSat1-related sequences showing the conserved region used for probe design.

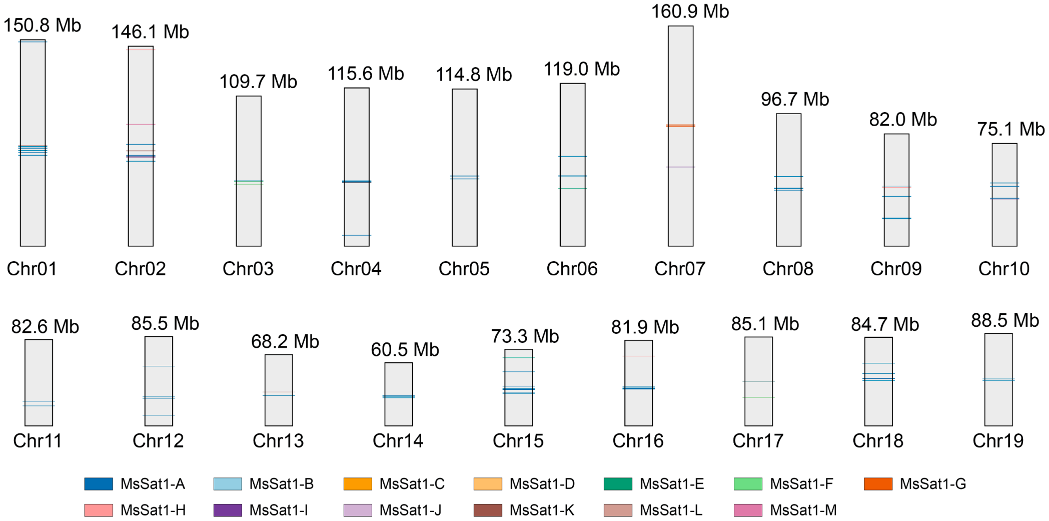

Figure S5. Genome-wide distribution of MsSat1-derived variants. Chromosomal distribution of MsSat1-derived variants across the *M. sinensis* genome. Different colors indicate different variant types.

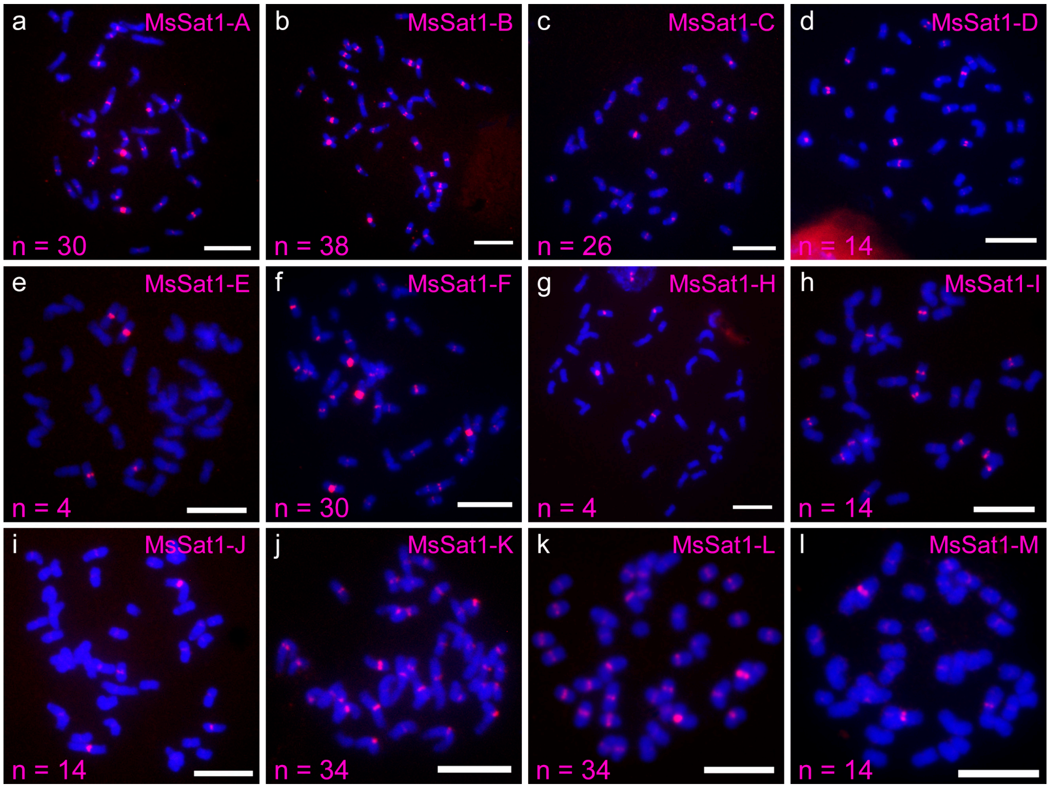

Figure S6. FISH validation of MsSat1-derived variants to *M. sinensis* accessions of MH149**.** Representative FISH signals of MsSat1-derived variants on metaphase chromosomes. The number of detected signals is indicated in each panel. Chromosomes were counterstained with DAPI, Bar = 10μm.

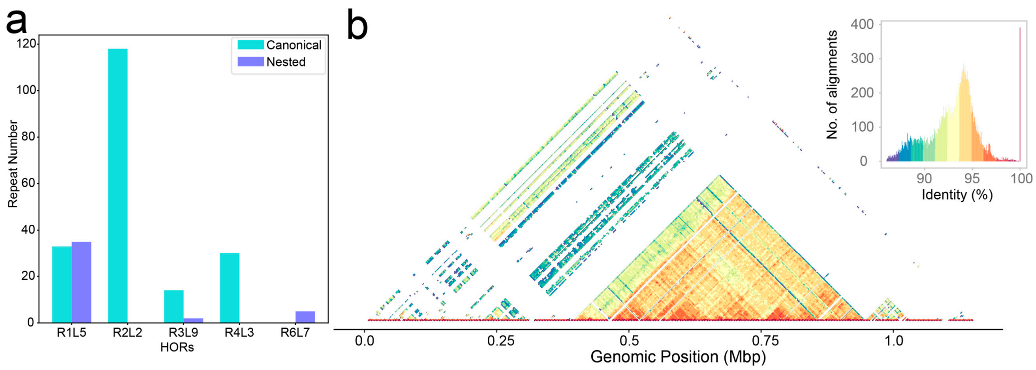

Figure S7. HOR organization of MsSat1 arrays. (a) Numbers of canonical and nested repeats in different HOR types. R indicates the repeat-pattern type and L indicates the number of monomers comprising the corresponding HOR unit. (b) Dot-plot visualization showing the organization of representative MsSat1 HOR arrays. Both axes show genomic positions along the same array. Colored points represent local pairwise alignments, with colors indicating sequence identity. The inset shows the distribution of alignment identities. Recurrent diagonal and off-diagonal blocks reveal the internal organization of canonical and nested HOR structures.

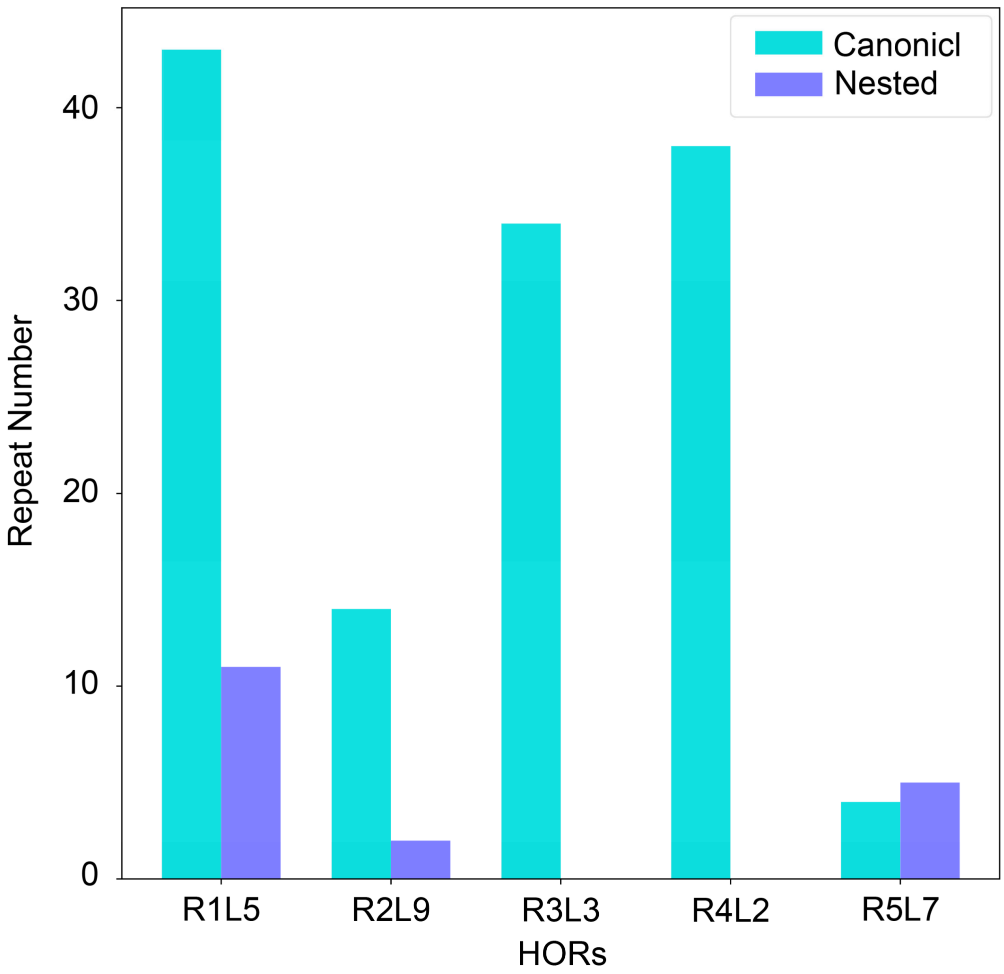

Figure S8. Quantification of canonical and nested HOR repeats. R1L5, R2L2, R3L9, R4L3, and R6L7 denote representative MsSat1 HOR arrays, where R indicates the repeat-pattern type and L indicates the number of monomers comprising the corresponding HOR unit. Bar plot showing the repeat numbers of canonical and nested structures among different HOR types.

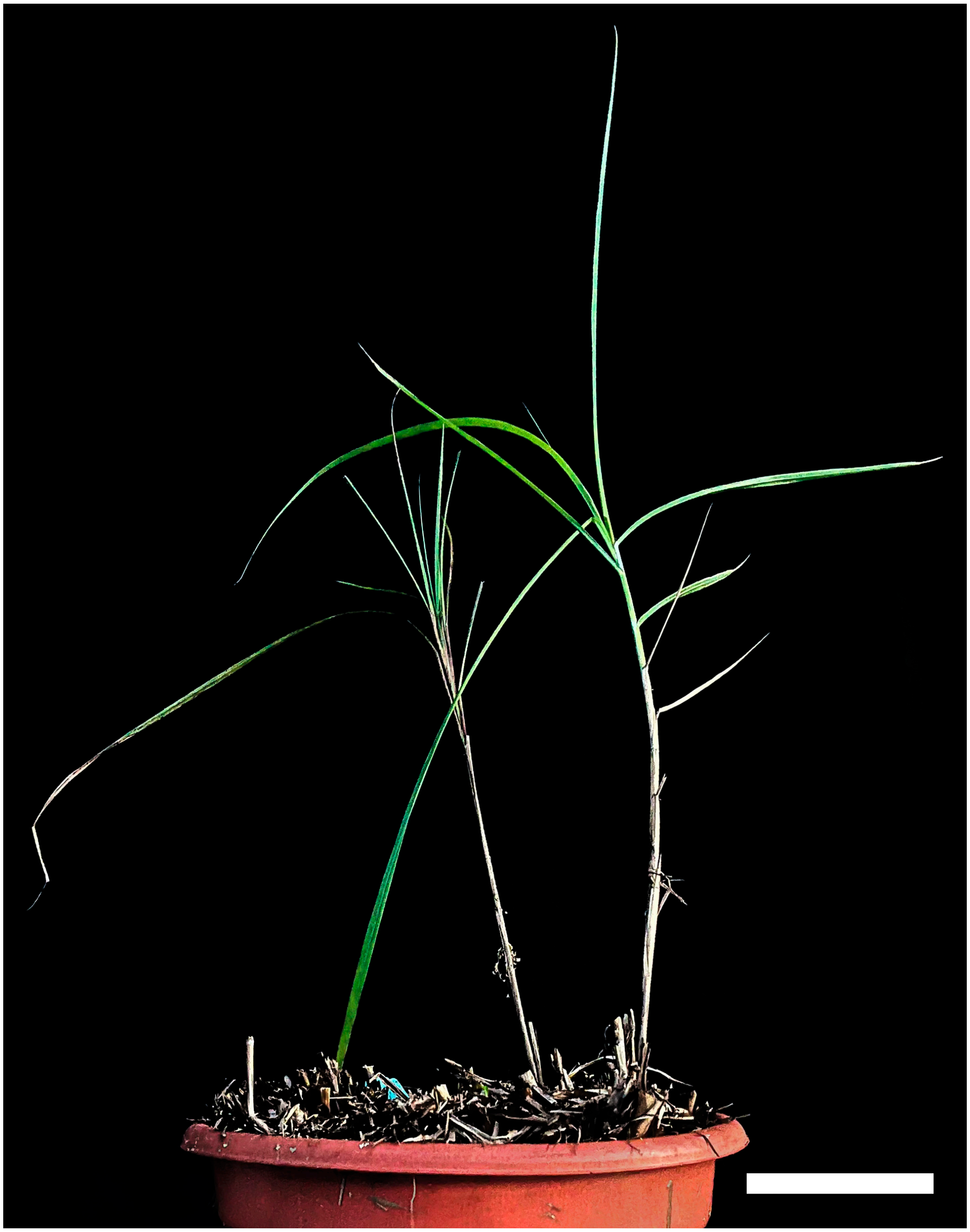

Figure S9. Phenotypes of *M. sinensis* accessions M142. Bar = 10 cm.
