# Supplementary material for: Fusion-Derived Centromeres Reveal Satellite Repeat Remodeling in Miscanthus sinensis
Source: Plants (Basel). 2026 Sep 18;15(18):2855. doi: 10.3390/plants15182855 (PMC13610342; doi:10.3390/plants15182855)
Supplement: Supplementary file 1 [file plants-15-02855-s001.zip › Supplementary Table.pdf]

Table S1. The information of representative candidate satellite sequence from first cluster.

Repeat	Repeat Sequence
MsSat1	ACGTTTGGCGCAAACCGTGCACCTATCTTGCACCAACACTAACACTATCTCCAAACGGACCGAAACGAGATTCCACATGACCCACGTCACCTAGGAGTTCCATCGGGTGCGTCCAAAATGATTTCTGAGCCTATGGT

MsSat 48

GTTGGAGCGGAGCGGCATGGCTGAGTGCAAGCCATGCGTGACTCCGATGGAGGAGCGGCTGAAGCTGACGAAGGCCAGCACCGCGGCGAAGGTGGATGCAACACTCTACCGGAGCATCATCG
GCGGTCTGCGCTACCTAGTCCACACGAGGCCGGACATTGCGTTCGCCGTGGGCTACGTCAGTCGCTTCATGGAGGATCCCAGAGAGGATCACTGGGCTGCGGTGAAGCGGCTACTGCGCTACGT
CAAGGGGACGGTGGATCAGGGGATCATCTTCCCAAAGACCGGCGGGAGTAGGCTGCAGCTCACTGTGTTCAGCGATGCAGACATGGCGGGGGACATCGACGGACGACGGAGCACCTCTGGCG
TGCTCGTCTTCCTCGGGTCGGCTCCAATTTCATGGCTGTCGCTGAAACAGAAGGTGGTGGCGCTATCTACGTGCGAGGCAGAGTACGTAGCGGCGGCCACAGCGGCGTGCCAAGTTGTGTGGCT
ACGCCGGCTGCTGGGCGAGCTGACCGGTGTGGAAGCTCACCCACCAGCACTGATGGTGGACAACCAGCCCGCCATCGCCCTCGCGAAGAATCCGGTTCTCCACGACCGGAGCAAACACATCGA
CGTGAAGTTCCACTTCCTCAGGGACTGTGTCGATGGAGGGCAGATCGTCATCGAGTTCGTCGAAACTGGTCGGCAACTCGCGGACGTCCTCACCAAGCCGCTCGGACGTCTTCGACTCACGGAG
CTGAAGGAGATGATCGGCATGGAGGGGGTACAAGGGTTAGCAGTAGGATTAGGGGAAGAATTGTTAGATAATCTACTGCTTCCTTGTGTGAACACACAGCAAGGGAAGGCGGCGCCGAAAAG
GCTCCCTGCTGTGGTACTGTAGCCGCTGCAGGTGCAGGCGCCGCACGGCTCACCTGCCGCACTGTAGCCACATGCAGGGACAGGCGCAGAAGTCAGTCCTGCTGTGTTATCTAGTCACTGTTGC
AGCATGTCAGCTGTACTAGGACTAGATGGATAGAGTTGTATAAATAGTTTGCCACTGCAACTCAGTAAAGAGAGTTCAGATTTGCCATCTCCTATACAGGGCTCCGGCCAACGCTGGTGCTTGT
ACTGTGTGTGTGCTCTGTTCTCCCTCTTCTTCTACCTCTAGCCATAGTGTGTGGGGACGGACAACGCTTGTTCGTGGTCGGCACAGCTAGTGGGTGCTCGGCAACACTAGCGGGTGCTCGGCAAG
ACTGGTGAGTGGTTCACTCACCTGAGCCGGTGATCCTGTGGGCCAACAAGTGGTATCAGAGCCGTACAAGCGCCGTCGCCGGACTAACCGCTGGCGATGACGGACGGTTCGGAGATGGGCGAC
AGCAGTGGCACTGCTGTGGCTGCCCAGCCACGACAGGAGGTCGTTGTGCGCACGGTGCGGGAGGTCAGCGGCACCAGTTGGCCGACGCTGACTCGCACCAACTATGGAGAGTGGTCGGTGACC
ATGAAGGTCAAGCTCAGAGCCCGACGGCTCTGGAATGCTATTGACAAGGGCACCGACAATGAAGAAGATGACATGTCAGCGTTGGAGGCTATCCTCGCTGCTGTACCGGCGGAGTACAGGGAG
CCGTTGGGGGCGAAGAGCTCTGCTAAGGAGGCGTGGGAGGCTATTGCGGCGATGCGCGTCGGTTCCGACCGCGCAAAGAAGGCGACGGCCCAGCTTCTGAAGCAGGAGTACGCCAACCTCAA
GTTCAAGGATGGTGAATCGGTGGAGGACTTCTCCCTCCGCCTGCAGACGCTCATCAGCAAGCTGAAGAGCCACGGCGTCACCATCGACGAAGAGGAGGCGGTCTCCAAGTACCTCCACTCCGT
GCCGGCAAAGTACATCCAGATCGCTCTCTCCATAGAGACGATGCTGGACTTGTCCACCCTCACCATTGAGGATGTGACAGGCCGTCTGCGGGCGGTGGACGAGCGCCTGGAGCAGGCGACAGC
AACGAAGGACAGCGGCAAACTGCTGCTGACAGAGGAGGAGTGGGCTGCTCGGAGGAACTCCGGGAAGGCAGCCTCCTCCAGCCGCGGTGGCGATGGCAAGCGCCGCGGCAAGGCTTCTTCGG
AGAAGAAGAAGCAGGTCGACCCCAACGCCTGCCGGCGCTGCGGGAAGACGGGCCATTGGGCAAGGGAGTGCCCAAATCGCAAGCAGGAGAAGAAGGCTGAGGCTCATCTGGCGCAAGCTGAT
GATGATGATGAGGCCACTATCCTGATGGCGACGTTCTGTGCACTGCACGACGTCGAGGCCGAGGAGAAGGAAGAGGTGACGACGGTGGAAGGACCTGGGAAGGCCCTGAAGGCTGTCAACCT
CGACGAACCACGCGCCCAAGTCCACCTCGGACGTGTGGGCGCCGACCAGGAGCAGCGGTGGTATCTGGACTCTGGTGCCAGCAACCACATGACGGGCTCCAAGGCAGCCTTCTCCGAGCTCGA
CGACGATGTTACCGGTACGGTGAAGTTTGGTGACGGCTCAAGGGTGGCTATCCAAGGGCGCGGCACCATCATCTTCAGGTGCCAGAACGGGGAGCACCGCGCGCTAACGGATGTATATTACAT
CCCGCAGCTGCGTTCAAGCATCATCAGCATTGGCCAGCTGGATGAGCGCGGTAGCGAGGTACTGATCAAGGACGGAGTCCTTAGGATCAGGGACCGGGAGCAGCGACTTCTTGCCAAGGTGAA
GAGGTCCCAGAACCGGTTGTACCTGCTCGACCTGAAGGTAGAGCAGCCGGTGTGCCTGGCGGCAAGGCACACCGAGGAACCGTGGCTGTGGCATGCCCGGTTCGGACATCTCAGCTTCGACGC
GCTTGGTCGGCTGGAGAAGATGGTCCGAGGGCTACCCCACATCAAGCACGGAGGCGAGCTGTGTGACAGCTGCCTGGCCGGGAAGCAGAGGAGGCTACCATTCCCAAAGGCGGCCAAGTATC
GCGCGAAGGACGCTCTCGAGCTCGTCCACGGCGACCTCTGCGGGCCGATCACGCCAGCTACAAACGGTGGTCGGCGGTACTTCCTCCTGCTCGTGGATGATTGCAGTCGCTACATGTGGCTGCA
ACTCCTGACGAGCAAGGACGAAGCGGCGGCGGCGATCAAGAAGTTCAAGACGCGCGCGGAGGCCGAGAGCGGCAAGAAGCTCCGCGTGCTGAGGACTGATCGCGGCGGCGAATTCACTTCGG

TGGAGTTCGCTGCGTACTGCGCGGATCAGGGTGTGGTGCGACACCACACCGCGCCGTACTCGCCACAACAGAATGGCGTGGTGGAGCGGCGGAACCAGACGGTGGTCGGCATGGCTCGATCCA
TGATGAAGGCCAAAGGCATGCCGGCAAGGTTCTGGGGTGAGGCGGTGACCACGGCGGTGTTCATCCTCAACCGCGCTCCCACCAAGGCCCTGAAGGGCAAGACGCCGTTCGAAGCTTGGTATG
GGCGCAAGCCGAGCGTGTCCTTCCTCCGGACATTCGGCTGCATCGGCCACGTCAGGAAGACGAAGCCGATCCTCACCAAGCTGGAGGACAGGAGCACACCGATGGTGTTCCTAGGCTACGCGG
AGGGTACCAAGGCGTACCGGCTCTATGACCCACGCGGAGACAAGGTGCTTGTCTCGCGCGACGTCGTGTTCGACGAGAAGGCGGCTTGGGACTGGAACAGTCCGAGCACGGGGGAAGCTGGCG
GCTTCACCAGCACCTTCGTCGTCGAGCACTTGGTCATCCACGGTGGTGGAGACGCTGGGGAAGAGGTGCCGAGCACTCCAGGTGCTGTGCCGACCACTCCGGCGGAACAGGGAACTCCATCGA
CGCCGATCGAGTTCGCCTCACCTCCAAGTGACATCACTGAGTTCGTGGATGCCTTCCACGAAGGTGAGGAGGTGCGGTTCCATAGGCTGGACGACATCATCGGCGGCACAGGGCCCTCAGGCCT
GGCGGGTCGGCTGCTCAATGACCCAGAGCTGCTTCTCGTCAGTGCAGAGGAACCACCCACGTTCGCGCTGGCCGAGCGCGATGGAAATTGGCGACGGGCGATGCTGGAGGAGATGAAGGCGAT
CGAGGAAAACGAGACTTGGGAGCTCGTCGATCCACCTCCAGGATGCCGTCCGATCAGCCTGAAGTGGGTGTACAAGGTCAAGCGGGACGAGCTCGGCGCCATTGTCAAGCACAAGGCGCGCCT
CGTCGCCCGAGGCTTTGTCCAGCGTGAGGGCATCGACTTCGAGGAAGTCTTTGCGCCAGTAGCGCGCATGGAGTCGATCCGTTTGCTACTAGCTTTGGCAGCAGCAAAGGACTGGCGCGTCCAT
CACTTGGACGTTAAATCGGCCTTCCTCAATGGCGAGCTGGCGGAGACGGTCTTCGTCAGGCAACCTCCAGGTTTCGCCGTCAAGGGAGCGGAGCACAGGGTGCTCCGACTGCGCAAGGCGCTCT
ACGGGCTGCGGCAGGCCCCACGAGCATGGAACGCCAAGCTTGACGCCACGCTGGGCGAGCTTGGGTTCACGCGGTGCGCAACCGAGCACGCGCTCTACACGCGGCGACGGGGGAAGGAGGAG
CTCGTCGTCGGCGTGTATGTGGATGACTTGATCGTCACCGGCGCGCGTACGGAGGACATCAACAGCTTCAAGCGCGAGATGGCGGCTCGTTTTCGAATGAGCGATCTCGGCGCACTCTCCTACT
ACCTCGGCATCGAGGTGAGACAGGGGAAGGAGGAACTCACGCTCGGTCAGAGCGCGTACGCCTCGAAGCT

	CCGGTCTCCGCGCGCCGCAAGGCGCGCCCCTCGGGGCGCTGCGCTGCAACGGCCTGCGAGCTCCCCATCCGACCCGTCTTGAAACACGGACCAAGGAGTCTGACATGCGTGCGAGTCGACGGG
	TTCTGAAACCTGGGATGCGCAAGGAAGCTGACGAGCGGGAGGCCTTCACGGGCCGCACCGCTGGCCGACCCTGATCTTCTGTGAAGGGTTCGAGTTGGAGCACGCCTGTCGGGACCCGAAAGA
	TGGTGAACTATGCCTGAGCGGGGCGAAGCCAGAGGAAACTCTGGTGGAGGCTCGAAGCGATACTGACGTGCAAATCGTTCGTCTGACTTGGGTATAGGGGCGAAAGACTAATCGAACCATCTA
	GTAGCTGGTTCCCTCCGAAGTTTCCCTCAGGATAGCTGGAGCCCATTACGAGTTCTATCGGGTAAAGCCAATGATTAGAGGCATCGGGGGCGCAACGCCCTCGACCTATTCTCAAACTTTAAAT
	AGGTAGGACGGCACGGCTGCTCCGGTGAGCCGTGCCACGGAATCGGGAGCTCCAAGTGGGCCATTTTTGGTAAGCAGAACTGGCGATGCGGGATGAACCGGAAGCCGGGTTACGGTGCCGAAC
	TGCGCGCTAACCTAGAACCCACAAAGGGTGTTGGTCGATTAAGACAGCAGGACGGTGGTCATGGAAGTCGAAATCCGCTAAGGAGTGTGTAACAACTCACCTGCCGAATCAACTAGCCCCGAA
	AATGGATGGCGCTGAAGCGCGCGACCCACACCCGGCCATCTGGGCGAGCGCCATGCCCCGATGAGTAGGAGGGCGCGGCGGCCGCCGCAAAACCCGGGGCGCGAGCCTGGGCGGAGCGGCCG
	TCGGTGCAGATCTTGGTGGTAGTAGCAAATATTCAAATGAGAACTTTGAAGGCCGAAGAGGAGAAAGGTTCCATGTGAACGGCACTTGCACATGGGTAAGCCGATCCTAAGGGACGGGGTAAC
	CCCGGCAGACAGCGCGATCTCGCGTGTTCCCCGAAAGGGAATCGGGTTAAGATTTCCCGAGCCGGGACGTGGCGGTTGACGGCGACGTTAGGAAGTCCGGAGACGCCGGCGGGGGCCTCGGGA
	AGAGTTATCTTTTCTGCTTAACGGCCTGCCAACCCTGGAATCGGTTCAGCCGGAGGTAGGGTCCAGCGGTCGGAAGAGCACCGCACGTCGCGCGGTGTCCGGTGCGCCCCCGGCGGCCCTTGAA
	AATCCGGAGGACCGAGTACCGTCCACGCCCGGTCGTACTCATAACCGCATCAGGTCTCCAAGGTGAACAGCCTCTGGCCAATGGAACAATGTAGGCAAGGGAAGTCGGCAAAACGGATCCGTA
	ACTTCGGGAAAAGGATTGGCTCTGAGGGTTGGGCTCGGGGGTCCCGGCCCCGAACCCGTCGGCTGCCGGCGGACTGCTCGAGCTGCTCGCGCGGCGAGAGCGGGCCGCCGCGTGCCGGCCGGG
MsSat	GGACGGACCGGGAACGGTTCCTTCGGGGGCCTTCCCCGGGCGTCGAACAACCGACTCAGAACTGGTACGGACAAGGGGAATCCGACTGTTTAATTAAAACAAAGCATTGCGATGGTCCCCACG
49	GATGCTGACGCAATGTGATTTCTGCCCAGTGCTCTGAATGTCAAAGTGAAGAAATTCAACCAAGCGCGGGTAAACGGCGGGAGTAACTATGACTCTCTTAAGGTAGCCAAATGCCTCGTCATCT
	AATTAGTGACGCGCATGAATGGATTAACGAGATTCCCACTGTCCCTGTCTACTATCCAGCGAAACCACAGCCAAGGGAACGGGCTTGGCGGAATCAGCGGGGAAAGAAGACCCTGTTGAGCTT
	GACTCTAGTCCGACTTTGTGAAATGACTTGAGAGGTGTAGGATAAGTGGGAGCCTTCGGGCGCAAGTGAAATACCACTACTTTTAACGTTATTTTACTTATTCCGTGGGTCGGAAGCGGGGCAT
	CGCCCCTCCTTTTGGCTCTAAGGCCCGGCCTCGCCGGGCCGATCCGGGCGGAAGACATTGTCAGGTGGGGAGTTTGGCTGGGGCGGCACATCTGTTAAAAGATAACGCAGGTGTCCTAAGATGA
	GCTCAACGAGAACAGAAATCTCGTGTGGAACAAAAGGGTAAAAGCTCGTTTGATTCTGATTTCCAGTACGAATACGAACCGTGAAAGCGTGGCCTATCGATCCTTTAGACCTTCGGAGTTTGAA
	GCTAGAGGTGTCAGAAAAGTTACCACAGGGATAACTGGCTTGTGGCAGCCAAGCGTTCATAGCGACGTTGCTTTTTGATCCTTCGATGTCGGCTCTTCCTATCATTGTGAAGCAGAATTCACCAA
	GTGTTGGATTGTTCACCCACCAATAGGGAACGTGAGCTGGGTTTAGACCGTCGTGAGACAGGTTAGTTTTACCCTACTGATGACCGTGCCGCGATAGTAATTCAACCTAGTACGAGAGGAACCG
	TTGATTCACACAATTGGTCATCGCGCTTGGTTGAAAAGCCAGTGGCGCGAAGCTACCGTGTGCCGGATTATGACTGAACGCCTCTAAGTCAGAATCCAAGCTAGCAACCGGCGCCTGTGCCCGC
	CGCTCGCCCCGACCCACATTAGGGCGTTCGCGCCCCAAGGGCCCGTGCCATTGGCCCAGCCCGCCCGGCCGACGCGCCGCGGCGGGCCGCCTCGAAGCTCCCTTCCCAACGGGCGGCGGGCTG
	AATCCTTTGCAGACGACTTAAAACGCGACGGGGCATTGTAAGTGGCAGAGTGGCCTTGCTGCCACGATCCACTGAGATCCAGCCCCGCGTCGCACGGATTCGTCCCTCCCCCTCCACTCTCGCG
	GGATGAAGTCAGGCTGGGAGTCCGCGTGACCGTGGCCGACCCAAGTCCCCGGGTCAAAAACAGCAGTTCGGCCAAGGCGTCCTGGGCCAAAAACAGCAGTTCGGCCGAGGCGTCCGTGAGCA
	GCCCAAGTCCCCGGGAAAGCCGGGTTTCGTACGCGTGACGGATGTGCGGCTTGGGCAAAAATAGCAGCTCGGCCGAGCGACATCGGTTCGGCAAGCCGGTCCGCGCTCGGGCTACGGGGCGCA
	CACACGACTTAAGTGCCACCGCACCGTACACCACAGGGGCTAAGCGTGTATGCGCGGTCGGATGGACTTTTTTTTTTTTGCATGTGCGCGGTCGGATGGACTTGCGTGTAAGCGTGTGTGTGCGC

GGTCGGATGGACTCCCCGTGTAAGCGTGTATGCCCGGTCGGATGGACCTTGGGCGTGCATACCCGGTCCACCAGCTGTTCACCCACCGTACACACGACCGCGTGGTCGGATGGACTTGCGTGCG
TGTATGCGCGGTTGGTTGGATGGATGGCGACGAACGGCTAGGACTCGGACCGCGCGTGATGGATATGGCTCACGAGGTCACGGGAGAAGGACAAGATGACGTCGGGACAAACGAGCCCGAAA
CAGGACCCGGTCCCACCGGGGGTTGGCCCACGGGGCCGGTCCATCCGCGGAAAACAACGAATGCCGTGCGCCTCCTCGGCGGAAACCTGCGTGTCTTGCCGAAAACCTTTGCAAAACTACGTA
TCACATGGATGTCCTGAGGTCCTCCAAAACACTGGAAGGAACCATACAGAAAACTGACAGAACCTCCTGGTAGTGGGTTTGGCCCTCTGGCACATTAGGGGGTGATGTCCGGTTGCCTGAGGCC
TCGGGTAAAATGCTTCGAAACCAGTAGTGTTTCGATGACCCGCTAATGAGGCTCCACCGAATGTCGGTCACGCCCCCAGCGCCCGTGGTCGGCTGGCGGCCTGCCGTGGCTAGCTTCGGCTGGC
TGCAGCGGGCTGCTGGCTGCCCGGCTGGCCAGCGTGCGGCCATTAGTGGTGGCACGCTGTTGGACTGAAGTGGCACAAGTTTTGGTGGGCAGCTAGCTGGATTGGGTGGCCAGCGTGTTGTTGG
AACTGTTTCGGTGACTCGCGGCCTCCCTTGTCGGCCGGCAGCCTACGGTGGCGTGCTTCTATGGCGTGCCGCTGTGGGCAGCTGGCTGCCGTGGGTGTGTAGCTTGTTGTTGGAACGGTTTCGGT
GATGCGCGGCCTCCCTGGCCGGCCGGCAGCCTGTGTTGGCCTGCTTCTGTGGGCGCTGGCCCATTGATGGTTAAGTTTTGGTATCTCCCGTGCTCTGTGGACCTTCGGTTGCTATCCTCGCAAGCA
GACTCACCGTGCCTAGCGCGGAGTGGTGCTTTGGATGGCTTAACCGTCGTGGCTACGCTGGCGCATGAGTTGTTTTAGACCTGTGACTGCTTGGAGGACCCCCTACCGATGCGTGGCCGACTCCT
GGCGCCAGTGTCCGTTCATTCGTGCGGGCATGTTTTGCCTGCTGCATTGACAAGTGCTTGCATGCTCTACCTGTTGCATTGGAAGTTGTGCTCATTACATGATGTTTACGTCGAGAACATCCTCTG
GGTGTGGCTCGTCGGCTACAAAGCGCTTGTGGCGTTGCCTCGTGCTGCCGTCCGCCCATGTCGGCAGCACCAAGGACACCTCGCTTGTGCTCTTGGTCTTGGATGTGGCTGATGCTGAAGGCCGG
AGGGCGTCAGCCTCCCGTGCCTAAAAATTCAGTCCGTTTGGAAACAACGACAGCCAGCCCCTGGCCGATGCCTCGTGTGGAGGCTGGCGCTGGCTCGTCGATGAGGACGTGCTACCTGGTTGAT
CCTGCCAGTAGTCATATGCTTGTCTCAAAGATTAAGCCATGCATGTGCAAGTATGAACTAATTCGAACTGTGAAACTGCGAATGGCTCATTAAATCAGTTATAGTTTGTTTGATGGTACGTGCTA
CTCGGATAACCGTAGTAATTCTAGAGCTAATACGTGCAACAAACCCCGACTTCTGGGAGGGGCGCATTTATTAGATAAAAGGCTGACGCGGGCTCTGCTCGCTGATCCGATGATTCATGATAAC
TTGACGGATCGCACGGCCCTCGTGCCGGCGACGCATCATTCAAATTTCTGCCCTATCAACTTTCGATGGTAGGATAGGGGCCTACCATGGTGGTGACGGGTGACGGAGAATTAGGGTTCGATTC
CGGAGAGGGAGCCTGAGAAACGGCTACCACATCCAAGGAAGGCAGCAGGCGCGCAAATTACCCAATCCTGACACGGGGAGGTAGTGACAATAAATAACAATACCGGGCGCGTTAGTGTCTGG
TAATTGGAATGAGTACAATCTAAATCCCTTAACGAGGATCCATTGGAGGGCAAGTCTGGTGCCAGCAGCCGCGGTAATTCCAGCTCCAATAGCGTATATTTAAGTTGTTGCAGTTAAAAAGCTC
GTAGTTGGACCTTGGGCCGGGTCGGCCGGTCCGCTTCACGGCGAGAACCGACCTACTCGACCCTTCTGCCGGCGATGCGCTCCTGGCCTTAATTGGCCGGGTCGTGCCTCCGGCGCTGTTACTTT
GAAGAAATTAGAGTGCTCAAAGCAAGCCATCGCTCTGGATACATTAGCATGGGATAACATCATAGGATTCCGGTCCTATTGTGTTGGCCTTCGGGATCGGAGTAATGATTAATAGGGACAGTCG
GGGGCATTCGTATTTCATAGTCAGAGGTGAAATTCTTGGATTTATGAAAGACGAACAACTGCGAAAGCATTTGCCAAGGATGTTTTCATTAATCAAGAACGAAAGTTGGGGGCTCGAAGACGAT
CAGATACCGTCCTAGTCTCAACCATAAACGATGCCGACCAGGGATCGGCGGATGTTGCTTATAGGACTCCGCCGGCACCTTATGAGAAATCAAAGTCTTTGGGTTCCGGGGGGAGTATGGTCGC
AAGGCTGAAACTTAAAGGAATTGACGGAAGGGCACCACCAGGCGTGGAGCCTGCGGCTTAATTTGACTCAACACGGGGAAACTTACCAGGTCCAGACATAGCAAGGATTGACAGACTGAGAG
CTCTTTCTTGATTCTATGGGTGGTGGTGCATGGCCGTTCTTAGTTGGTGGAGCGATTTGTCTGGTTAATTCCGTTAACGAACGAGACCTCAGCCTGCTAACTAGCTATGCGGAGCCATCCCTCCGC
AGCTAGCTTCTTAGAGGGACTATGGCCGTTTAGGCCACGGAAGTTTGAGGCAATAACAGGTCTGTGATGCCCTTAGATGTTCTGGGCCGCACGCGCGCTACACTGATGTATTCAACGAGTATAT
AGCCTTGGCCGACAGGCCCGGGTAATCTTGGGAAATTTCATCGTGATGGGGATAGATCATTGCAATTGTTGGTCTTCAACGAGGAATGCCTAGTAAGCGCGAGTCATCAGCTCGCGTTGACTAC
GTCCCTGCCCTTTGTACACACCGCCCGTCGCTCCTACCGATTGAATGGTCCGGTGAAGTGTTCGGATCGCGGCGACGGAGGCGGTTCGCCGCCCTCGACGTCGCGAGAAGTCCATTGAACCTTAT

CATTTAGAGGAAGGAGAAGTCGTAACAAGGTTTCCGTAGGTGAACCTGCGGAAGGATCATTGTCGTGACCCTTAAACAAAACAGACCGCGAACGAGTCCCTCGTGCCGCCGAGCTTCGGCTCG
GCATTAGGTCCCCGAGCTCCGTCCCGGGGCGGAGGGGCCACAACAGAACCCACGGCGCCTTAGGCGTCAAGGAACACTTATATTGCCTTGCTCGGCGGGGCGGTCGGCCTGCCTTCCGCTCCCC
GCGCAGCGATGATATCTTAATCCACATGACTCTCGGCAACGGATATCTCGGCTCTCGCATCGATGAAGAACGTAGCAAAATGCGATACCTGGTGTGAATTGCAGAATCCCGCGAACCATCGAGT
TTTTGAACGCAAGTTGCGCCCGAGGCCTTCTGGCCGAGGGCACGTCTGCCTGGGCGTCACGCCAAAAGACACTCCCAACCCACCCAAGGTGGAGGGACGTGGTGTTTGGCCCCCCGCGCCGCAG
GGCGTGGTGGGCCGAAGTTGGGGCTGCCGGCGAATCGTGTCGGGCACAGCACGTGGTGGGCGACACTCAGTTGTTCTCGGTGCAGTGCCCCGGCAATCGGCCGGCGCATCGGCCCTAAGGACC
CTTGGAGCACCGCAGCGCATCGCCGCTCGGACCGCGACCCCAGGTCAGTCGGGACTACCCGCTGAGTTTAAGCATATAAATAAGCGGAGGAGAAGAAACTTACAAGGATTCCCCTAGTAACGG
CGAGCGAACCGGGACCAGCCCAGCTTGAGAATCGGGTGGCCTCGTCACCCGAATTGTAGTCTGGAGAGGCGTCCTCAGCGACGGACCGGGCCCAAGTTCCCTGGAAAGGGACGCCTGGGAGGG
TGAGAGCCCCGTCCGGCCCGGACCCTGCCGCACCACGAGGCGCCGTCAACGAGTCGGGTTGTTTGGGAATGCAGCCCAAATCGGGCGGTAAACTCCGTCCAAGGCTAAATACAGGCGAGAGAC
CGATAGCGAACAAGTACCGCGAGGGAAAGATGAAAAGGACTTTGAAAAGAGAGTCAAAGAGTGCTTGAAATTGCCGGGAGGGAAGCGGATGGGGGCTGGCGATGCGCCCCGGTCGTATGCGG
AACGGCTTCTGCTGGTCCGCCGATCGGCTCGGGGCGTGGACCGTTGTCGGCCGCGCCGGCGGCCTAAGCCCGGGGGCCCTAGGCGCCTCCGGCAGCCGTCGTCGGCACGG

MsSat76	ACGGTTTGCGCCGAACGTACCATAGGCTCAGAAATCAGTTTGGGCGCACCTGATGGTACTCCTAGGTGACGTGGGTCAAGTGGAAGCTTGTTTCAGTCCGTTTGGAGATAGTGTTAGTCTCGACGCAAG<br>ATAGGTGC
MsSat77	TCTACTCTCTACATACAACATGTGGTACTCCATGTTAAGATTTGGTATATTTCTAGATCCGTTTGCTATATTTAATTAATTTATTGCTTTTCAAGGAATTTTTTGGTATAAGTCAAATTTGAACTGCAAGTG<br>ATTCAAATAATAGAATAAAATGAGTAGAAAAATGATATTCATGTTATTGAGTCCAGTGTGAGGCCTTACCCAGGAAATGAAAAGAAATTTCGAACATCTTGTTCAGGAAACACGACCACGAACGTGTG<br>GCCGAATGGTTTTTAAATTCTAAAAAAAGCAAACGAAGTCTGAAAATCATGAGATTTGTCAAGATCTCATGATATCATACGTGGAGGCTATGGTAAAAAATTGAGAAGGTTTCGCACAATCTGTCATGT<br>ACGATGTTTACAAACCGAAGTATCTCAGAAGAAGAATCGTAGCGTTGAGAAGGATTCGGTAAGATTTGGAGTCAAAGTGACGATCGAATTGGGGTTTGACTTCAAAACTTTTTGTATAGGCAATAGAGA<br>ACATAGATTGATTCATGTGTAATTTTGGTAATTTTTTGGATCCGTTTGATAATTTTAATTTATTAAGTGCAATTATAGAATTTTAATTGATATACATTGAATTTGAGCTATAACTGCATGAAATAATAAGTT<br>TTTTTTACTTCTGGCCTTGAAACTTTT

MsSat91	CGAGATAAAGACAAGCGACCCAAGCCCGTTACGGTCCAGGGGTTCGAAGGCTGGGCCCCCAAGGGTTTCGACAGCCGCCCCAGGGCAACAGAGTCAGGGACGACTACGGGCGAGCCTATACATGGCC<br>GAGGCCCAAGCAAGCGAAACGCTTGGGACGCCCTAAGTCGTGTCCGAGACCGGCAGGGAGGTCTCCGAATGGGATCCCACCGTAGGGAGGCACCGAGCCACCGAGGCCCAGCGAACGGCCTCGGCAC<br>CCACTAGAGAAACCCTCTGGTACTCTTGGAGTGCGTCTCTGGACCGCTAGCCGACCCCTAGCGAACGGGGCACGGGCCTCCACTCGGACTTACCCGATAACAGCTCACCGGAAGTGCCACCGCTCGCGC<br>CCACCGAGGGTAGCCTGGCATATTCCACCCCTCCTTCCGAGCGAAAAGGAAGCGCGAGGGTCGTACAAAAAGCCAGGAGAACCCCTGACGGCCCTCTTGCTCCGTGCAGAGGCTAGGGAGCTCCTTCT<br>GCAAAAGACGCCGAGACCCCGCGACCTAGGCTCGCACCCGAGGGGGCCTCGGCCGACAGACCCTCACGCGCGAGGGGCGAACCAAAAGCCAGGGGGACCTCTGACCGCTCTCTCGCTCCGTGCGAGA<br>GACTCGGGGGCTCCTCCTGCAACTTTGCCGAGACCCAGCGGCTCAGGCTCGCACACGAGTGGGCTCGGCAAACAGCCCCCGTCCGAGCGAAAAGGACGTGAGGGAGACGGACAAAAAAGCCGGGAAG<br>ACCCCTGACCGCCCTCTCGCTCCGTGCGGAGGCTCGGGGGCTCTTCCTGCACC
MsSat98	ACAAAGCTGAATAAGTATCCCGAGTACTTGTCTTAGTTGCTGGCCTATTCACGTTCCAAGTACCAGATGTATCAAGTGATGTGAACAGCAGAAATATGATTAGGAACAAGGCAGAAATCAGCACACGCA<br>AACACAGCTCAAATGGCGCTCTCTATGTGCTCCTCTAGATATTGTTCCACTTTTGCCCCTCTCTTTTTTCTCTATTTTTGGGCTGTCGTTGTTTTTTTGGGATTCTTTGACTTTTTCTTTTTATTTTTTTGATAT<br>TTTTTCTTCACTTAGGAGCACAAAAGAAGTAACCACAGAAAATATGAGCTTAAACAAGTGAAAGGCGTGGCCTGTGGAAAATTCAGAAGACGTGCTCAAAATTGACAAAAAGCTTGTGACCACGAAAA<br>GAGGATCTTGTGACCAGTTTTTGGCCAAAATAAAATTTTCTAAACCCG

MsSat126	TCTGCCACCGCCGCTTCCGCCTCCGCTCTGGCTGCTGCCAGCTCCGCCGCTGCCAGCCTCGACGCCCTTGCCGCCGCCGCAGCGGTCTCTGCCGCCGCTCGCTCGCGTTCCTCTGCCGCGGCAAGTTCGGC<br>CTCCTGCCGACGCCGCGTGCTCGAGGCGACCGAGCGCTGAGACTGCCCTGCGGACATGACGCGCTACCGGGGGGCTGCTGCGTGGGGAGAGGGCTGCTTCAGACGAGCTGGGAGAGGAGTGAACAGG<br>AGCGGCCGGAGGAGCTGCTGCTGCCAACAGCTGGGGCTGTTGTGGGGCTGGGAGAGAAGATGAGCAAGAGATGCTCAGGCTACAGGATAATACGGCTCTGATACCAGTTGTTAGTCGCTGAATTCTCA<br>CTCTTGGTAGTAGGAGAATTCTTACTCTCATCGAGAGAGGATGACACTAGGAGTTGGGGCAATTTTCTTGTCTATTTCTCACACAAGCTCACACAAATGCCATACCAACCTGAGGGGTTGGGGTTACATA<br>TTTATAGGCTGCTAGCCAGCCAAGCATATGCCAAGATGCTAGTCTAAGATGCTAATATGCTGTCCTAGCTAAGATGCTGTCCTCTAGTCTAAGATGCTGTCCTAAAAACAGAAAAACAGCCACAAGGAC<br>CATGTGAGCAGCACAGCCCCACAAAGACCAGCCACACATGAGACTTATCCATCATTCTCCCCCTAAGTCTTGTGCGTCGTCTTGTGGGAGAGTTGAACCATCCCGGTCCTGGAGCAGAGCTCAAGGAAC<br>TTGATCCTCCCAAGGGGCTTGGTGAGCAGGTCCGCAAGCTGGTCCTTGGTGTTGATGTAGCTCGCCTTGATGCTCCCTTCCTCCAAACAGCCTCGGATGAAGTGGTACCTCACCCGGATGTGCTTGCTGC<br>GTTCGTGGAACACGGGGTTCTTTGCCAGGGCCAGAGCGGACTTGCTGTCCACCCTGAGCTCCACCGCTCTAGTGTCTCTGCCGAGGAGATCACCAAGCAGTCGAGCGAGCCAGAGCGCCTGAGTCGAAG<br>CGGTGGAGGCCGCTATGTACTCGGCCTCGCAGCTGGACAGGGCCACCACCTGCTGCTTGACCGACTGCCAGCTAACGAGGCACTTGCCGAGGAAGAAGAGGATCCCGCTCGTGCTCTTGCTGGTGTCGA<br>TGTCGCCGGCGTGGTCGCTGTCGCTGTACCCGACGAAGTGTGCCGCCCCAGGGCACCTAGGGTAGTAGAGGCCGTGGTCGAGAGTCCCCGCAACGTAGCGGATGATCCTCTTCACAGCCTGCTGGTGCT<br>CCGTCGTCGGTCGCTGCATGAACCGACTAACGTAGCCGACGGAGAATGCCAAGTCCGGCCGTGTGTGGGCGAGGTAGCGAAGGCTCCCCACAAGACGCCGGTACTGCGTAGCGTCCACCTCCTCCGTCG<br>TGCTGTCGCGGCTCAGCTTCAGCCTCTCCTCCATCGGAGTGAGAGCTGGGTTGCAGTCGGTGAGCCCAGCTAGCTCAACGACGCGCTTGGCGTAGGCGGTCTGTCGAAGCGTGATCCCGGAGTCATCCT<br>GGTGCACCTCGATTCCCAGGTAGAAGGAGAGAGGCCCCAGGTCACTCATCTGGAAGGTGGCCTTCATCTCTTCCTTGAATGCCGCCACCTCCGCATCCTTGGTGCCGGTGATCACCAAGTCGTCGACGTA<br>GACACCCACCAGCAGGGCATTTCCTCCATTGCCCCGTCGGTAGATGGCCGCCTCGTGCGGGCTTTGCTCGAAGCCCATCCCCTTTAGCGTGGAATCCAACTTGGCATTCCACGCCCTCGGTGCCTGCCGC<br>AAGCCATAGAGGGCCTTGCGCAGGCGGAGCACCTTGCCCTCCTTGCCGGGGATCGCAAATCCCGGCGGCTGGTGCACGTAGACCTCCTCCTTCAAGTCGCCGTTAAGGAACGCCGACTTGACGTCCATG<br>TGATGAACACGCCAGCCCTCCTGGGCAGCTAGCGCAAGGAGGAGTCGCACGGACTCCATCCGTGCCACGGGAGCAAAGGCGTCGTCGAAGTCGACCCCCTCCTGCTGCACGAAACCTCGTGCCACCAA<br>GCGAGCCTTGTGCTTGACGATGGCGCCGGCTTCATCCCTCTTCAGCTTGTACACCCACTTAAGGGTGATCGCGCGGTGACCACGAGGAAGGTCAGCAAGCTCCCAGGTGCGGTTCTTCTCAACCGCATCC<br>ATCTCCAACTGCATCGCGGCGCGCCATGCCGCGTGTCTCTCGGCCTCTGCAAACGACCGAGGCTCGCCGTCGTCACACGCAAGGTGCAACTGCGCCTCCAGGTCGTGAGGCACCGGTCCCGGCACCGGC<br>TGGTCGCCGAGAAGGTTCTCCATCGTACGGTACCGCAACGGCTCGCCGTCGTGGTACGCGTCGATGCGCTCCTCGTCGTGAGAGAGCGGAGTAGCGAGCTCAACCGGGCTGTGCTCGACACGAGCTGGT<br>GGTGGAGTAGACGTGCCCGGAGTGGTGCCTGTCGGTGCTGGAGTGCGTGGCGTTGCCGGCTGTGGTGGAGCCGGCGAAGAACTCATCGCAGCCGAGGTCCTGGCTGGAGAGCGTGGTGCTGTCGGAGT<br>AGCAGGCGCCGGGGTCGGTGGAGGCTCGGGGACTGGGGTAGACGCGCTCGCCGAAGAAGAGCTGCCTACTCCCCCAGCTCCCTCGAAGTGGACGTACTCGACAGTGAAGTCGTCGTACGTCGGAGCCG<br>AGCCGTCGTCCACCGCCTTGTCCCACGCCCATCCTCGCCCTTCGTCGAACACAACGTCGCGCGCCGTGCGCACACGCTGTGTCTTCGGGTCGAGGATGCGGTAGGCCTTCGAGCCCTCCGCGTAGCCGAT<br>GAACACTCCCGGAGTGCTCCTGTCGTCGAGCTTGCTGATGTGGCCAAGCTCCTTGGCGAACGCGAGGCAGCCGAAGACCCGCAAGTGGGAGACCGCCGGCTTGCGCCCATGCCAAGCCTCGTACGGCGT<br>CCTGCCGTCGAGCGCCTTGGTAGGCGAGCGGTTGAGGATGTAGACGGCCGTCACCACCGCCTCTCCCCAGAAGACAGCCGGCATCCCCCTCTGCTTGAGGAGGGCCCGAGCCATCCCCACAACCGTCTG<br>GTTGCGCCGCTCGACGACGCCGTTCTGCTGCGGGCTGTACGGCGCGGAGTAGTGGCGCTGAATGCCCTCGTCAGCGCAGTACGACGCGAATTCAGCCGCCGTGAATTCGCCGCCGTTGTCGGTGCGCAG<br>CACGCGCAGCTTGCGGCCGCACTCCGCCTCCGCAGCAGCCTGCGCGCGCCTGATGGCGTCCGCAGCCTCTCCCTTGCTGCCGAGGACCATCACCCACATGTAGCGGGAGAGGTCGTCGACGAGCAGCAG

GAAGTAGCGTCGTCCTCCCGGTGTGGCCGGTGTCACCGGGCCACACAAGTCCCCGTGCACAAGCTCGAGCCTCTCCTTGGCTCGAAAGCTCGCCCGCTGGGGAAAGGGGAGTCGCCTCTGCTTCGTCAA
CACGCAGACGTCGCAGAGCTGCTCCACATGGTCGAGGCACGGCAGGCCTCGCACCATCTCCGTGGCACTGAGCCGCTTCAGGGCCTCAAAGTGAAGGTGCCCGAAACGCTCGTGCCACTGCCACGCCTC
GTCGTCCCGACGAGCAGCGAGACAGAGGGGTTGTGCCACCTGCACGTTAAGGACGTAGAGTCGATTTGCGCTTCTGGATACCTTGGCAAGAAGGCGACGACGACGATCCCAAATCCTCATGACTCCATC
CTCAACCACCACGCGCGAACCGTTCTCATCCAGCTGTCCCAAGCTGATGATGGAGTTCCTCAACGCGGGGATGTAGTAGACTCCGGTGAGCAGCCTGTGCTCACCAGACACGGCGGTGAAGATGACGGA
GCCGACGCCCTTGATCTCCACGCCGGAGGCATCCCCAAACTTGACGGAGCCTCGGACGCTAGAGTCAAGCTCGGTGAAGAACTCCCGTCGACCGGTCATGTGATGGGTGGCGCCGGTGTCGAGGCACC
ACCCGTCAGTCTTGTCGTTGCCGGAGCTGTCGCCGAGGAGGGCGTGTGCTTTTGGCTCGTCAAGGTGGAGGAGAGCCGCTGCGGCCGGTGCCGCTGGAGGTAGCTCGATGCTGGCATGTGCCATGAACA
GAGCCGGCTCCTCCTCCTCCGCCTGTGCGACGTGGGCCTGGCCGCGTCGTGGCTGTCGACAGTCCTTGGCCCAATGGCCAAGCTGGCCGCAGTTGCGGCAGGTGTCGTCTCGTGCCGGCTTGTGCCTGCC
GGCGGCGCCGCCCTGGGCGCCTCCGCGGGCATCACCCTCGGCACGTCCTCGCGCCCCGGCCTGGGCGTCTCTGCGCGCCTTGCGTGGCTTGCCACGCTTGCGGCCGCCTGTCGCGGAAGAAGGCTCCCC
CTTCTTCCGGTCACCTTGGCTGGCAAGCCACTGCTCCCGAGTGAGAAGGAGCTTCCCGCCAGTGGTGATGGGCCCCGAGAGGGACTGTGGCTCATCGCTGTCGACGACCTTGAGGCGACCTATCGCCTC
CTCGATCGACATCGTGGAGAGATCCAGCAGAGACTCGATCGAGCGAGCCATCTGCTTGTACTTCTCGGGGACGCAGCGGAAGAGCTTTTCGACAGCTCTCTCCTCGCCGTAGGTGTCATCGCCGAACTG
CACCATCTTCTGCAACAGAGTGTTGAGACGGAGAGCAAAGTCATCAACGTCCTCACCTGGCTTGAAGGCCAGGTTCTCCCACTCCTTGCGAAGTGCCTGCAGTGTGGACTTGCGGGCGCGGTCGCTGCC
GATGCGTGCCGCAGCGATGGCGTCCCAAGCCTCCTTGGCAGTCCGCTTGCTGGTAAGCGAGAACTGCATCTCGGGCGGGACTGCAGCGATGAGAGCATCCAGCGCCCGTCGATCTAGGTCGTAGTCGAC
GTCGCCGTACCGGACTGCCTCCCACATGTGCCGCACCTGGAGCTTTACCCTCATCACCGCAGCCCACTCGACGTAGTTGGTCTTGGTGAGGGTAGGCCACCCACCGCCGGGACCGACGTCCCTGACAAC
AGCCTGGAGCCCGTGGTAACCACGGTACCGATCCGGGGAGAGAGAGCCACGCTGCCTGTGAAGGCCGCGCTCTCCATCGACCCGTCCGCCACCGTTGCCGTGCGCGCCTCCTCCAGGAGCGCCGCCAGC
GCGTCCGCGCCTGTCTGGGCTGCCGCCACGCTCGTGGGCGTGCGCGGCTGCCCACTGCGCCGCCCGCGCTCGCGCTGCCTCCCTCTCTAGCAGCTCGAGGTCCGCGTCGGCGCTGTCGTCAGCGGAAATG
GAGCTGCCGATGCTGCCGCGCAGAGCCTCGACCTCC

MsSat167	GAGCCATTTAGAGCAAGGGGGTTGTCGCACGAAATAAAAACTGAGTCCCCTAAAGGCCTAAACGAGCTCGGTTGTAGAAAATGATGCGTTTTGTCACCATGGACTCGATTTCTCTCAGGGCCCTAGGCTCAAATTGATGACTAACTAGTTCGTGAAGAGTCTAGAGTAGTGTACGTGCCGACCTG
MsSat173	CATGGGTAAGCGATGTAATTGATACTAACATCATCTACGCTGTCTAGCCTAACTTCCTGTGTGCCACTCCTGCCTTACCCAGCTCGTTTCGGAACACGGGTGGACCCAGACATCTTCGACGGAATTGCAAGATTTCACCGAAACAAACGCTGCTTTGTGGACTATGATAAGGGATTTTTTAGAAAAATCTCTAGGGGCCCACTTTTGCCAATTCAGGTTCTCTAGGGTGAGGCCTGTAATTCTGCAGCCATGAAAAAAAACCTAGCTCGATTCCATGTCCGGAAGGCAAAGATCTCAAGGTCTTAAATTTGCCCTCAAATCTGCATCGGCAGTCCCCCGGTGTCGCGGCCACCAGTGTACCTTAACGC

MsSat178

AGCTATTGCTAGAATGATCCGGATAGATTTAAGCATCGCTACCGGCGAGAAAGTCTCGTCGTAGTCAATTCCTTGAACTTGCCGAAAACCCTTTGCAACAAGTCGAGCTTTATAGATGTGAACGTTTCCA
TCCATATCCTTTTTCTTTTTATAGATCCATTTGCACTCTATGGGTCTAACCCCATCAGGCGGGTCAACCAAGTTCCAAACTTGATTGTTTCCCATGGAATCTATCTCGGATCTCATGGCACTTTGCCATTTC
TCGGAATCTGGGTCCATCATTGCTTCCGCATAAGTCGCAGGTTCGTCATCGTCTAACAATAACAAATCCCCACGCAACTCATGGATTCTTGCTGACCTTCGTGGTTGTGGCGGTGTTTCTATTGCCACGGG
TATCTCAACTTGTTCTGCTACATTAGCATCACTCGTAGAGTCCTTCCCAACTGGCTCATCTTGAACTTCTTCAAGATACACCTTCTGTCCACTTTTCTCTCTTTTGAGAAACTCTTTCTCTAAGAAAACACC
GTTCCGAGCAACAAACACTTTGCCCTCTGATCGGTTGTAGAAGTAATACCCTAAAGTTTCCTTCGGATATCCCACGAAAAAGCATTTGTCTGATTTGGGTGTTAGTTTATCTGTCATAAGTCATTTGACAT
AAACTTCACAACCCCAAATTTTCAGAAAAGACAAACTGGGACTCTTACCAGTCCACATCTCATGTGGTGTCTTAACTACTGACTTAGATGGTACCCTATTCAATGTGAAAGCGGCTGTTTCTAGAGCGTA
TCCCCAAAATGATAACGGTAGGTCTGACTGGCTCATCATAGACCGAACCATGTCCAACAAAGTCCGATTACGTCGCTCGGACATGCCATTTCTCTGAGGCGTTCCAGGTGGCGTAAGCTGTGGAACAAT
TCCGCAACTCTTTAGATGATTGCTAAACTCGTGGCTCAAATATTCGCCTCCACGATCAGATCGCAAAGCCTTAATTTTCTTGCCACGTTGATTCTCTACTTCACTCTGAAACTCCTTGAACTTTTCAAATGT
CTCAGACTTATGTTTCATTAAGTAGACATAGCCATATCTACTAAAGTCATCAGTGAAGGTTATGAAGTATTGGAATCCGCCTCTTGCTGTCGTACTCATTGGTCCGCACACATCAGTATGTATGAGTTCCA
GCAAGTCTGCCGCCCTCTCAGGAAAACCTATGAAAGGCGTCTTGGTCATCTTGCCCAGCAGGCAAGCCTCACATGTCTCGTATGATTCAAAATCAAACGAAGTTAAAAGCCCATCAGAATGGAGCCTCT
TCATGCGATTCTCACTTATATGACCTAAACGACAATGCCACATATAGGTAGGACTTAACTCATTAAGCCGAGGCCTTTTAGCACTTATATTATAGACAGGAGCATCTTCAAGATTTAAAATAAATAACCC
ATTCACAATGGATGCAGAAGCCACAAACATGTCATTTTTAGAGATCACACAACCATTGTCTTTACTCGCAAATGAATAACCATCCTTCATCAAACATGAAGGTGACACAATGTTTCGACTTAAACTAGG
AACAAAATAACAATTATTCAACTCCATAATAAATCCTGACGGGAGGTGGAGTTGCATCGTCCCGACGTTCAACGCAGCAACTCTTGCATTATTGCCCACGCGGAAATCAACTTCTCCCTTTTCCACGCTT
CTACTTCTTATCATTCCCTGCATCGTATTGCAAATATGAGCAACCGATCCGGTATCAAATACCCAAGAATTAATATAAGAATTAGCAAGGAAAATGTCTGTAACATAAACAACAAGTGTACAAGCTGCG
GGAGTACCTTTACTGCCGCGATCCTTTATGGATTCTAGGTACAGCTTGCAGTTCCTCTTCCAGTGACCTTTCCCATGACAATGAAAGCACTCAGCATCAGCAGGTGGTCCAGCCTTGGGCGCAGGTGGGT
TTGGCTTAGAGATCTCATCTTTAGCCTTGCCCTTTTTCTTCTTCCAAGAATTGCCCTTCTTCTTAAACTTAGGCTTGTTTTGGACTGCCATCACATGGCTACTGCCAGCACCTTTCTTGATATCAGCCTCTGT
TGTTTTAAGCATGCCACATAATTCATTCAAGCCCTTCTCCGCCCCATGCATATGGTAGTTCGTGATGAAATTTCCATAGCTGGGCGGAAGAGACGCGAGAATAAAATCAGTAGCTAATTCAGGGCCAATT
GGGAAGCCCAGCTTCTCCAACCTCTGAGTGTAACCAACCATTTTGATCACATGTGGCCCAACTGCTGCGCCCTCTGCTAGCTTGGTTTCAGCAAAAGCCTTTGAGACATTGAACCTTTCAGTCCTGGCTTG
AGTCTGGAACATATCATTGAGCGCCACGATCATATCGTGCGCTGCATGGTTATTGTCAAACTGCAACTGCAGATCTGGTTCCATACAAGCAAGCATAAGGCAACTCACTTCAAGATCAGCATCACATGC
TTTCTTGTAAGCGTTTTTCTCTGCAGCAGGTGCATTATCAGCAGGCTCATCTGGTAATGGGGTGTCTAGAATTTCTTCCTTTTTCTCAGCCCTGAGAACAATTCTCAGGTTGCGGATCCAATCCGCATAGTT
TGTTCCATTCAACTTATCTTTCTCAAGAATTGAACGCAAAGTAAATGGTTGATTGCTAGGCGCCATTTATCTACAACAAAAGTAATGCAAAATACTAAGACAATGTATCCAAGACAGAGTAAACAATAT
TAAACCTTTAATAGAATCTACTCCCACTAAAATCAATATCCCTCTATTGAAACTTAGTGATTCAAGACCCACAACTAACAAGTCTACTAGTGAGCTTTAGCATCACCGCTAGAAGACATGGTAGATTGGT
AAGCAACTCTTTGCTAATCATATCACATATGACTCTTGTTGTTGGGTAGCATCTCTATGCTTTGGTGCCCAACTACTCATGCTCCAAGGTCCCTAACCGTTAGGATGACCTTGTCCAAGTAACCAACCCTT
CTGCTGTAAGTATCCGATACGAACCTGTCTAGTCAAGGAAAACCAGTGGCACCCTAATTTCATAGACCCACCACCGATTGTACAAGACACATGACAGTGCAAGGTTTGGGGAAGCATTTTAACTTAAAC
ATTGCCGAGGGATCGTTCTACTTCACCATCGCATGTAGACAAAAACATTATCGCACGTGAAAGTAGAACATAGTAAGAACGACATTAACACAGATATGACATGGTATAGCCCAATGTTCCTTTGGGGAT
CTCCATCTCCATCGAACTGTTCCTCATGATGATCTCCATCTCCATGATCCATGTATGCCATCCTTCAGTGATGAGTCCACCAAGACTAGCTATCACTAACGCAAGGCAGTGAAGAAAATTACATAGTCGC

GGATAGGATCATCACAGTTTGGCACGCAGGCCATTACATCAATTTGACAATATCTTAGTGGCTCCAGCCATATTGTCATACTCACGACATGCAAGCCATGAATTAATTACATACATGCATCACATACACA

TAAGGGCTATACCAGTCACAAATTCCTGCAAAACAGAGTTAACCGATTCCGACGTCTAATCTTAAAACGCCGAAAACACCATCTTCCCGGCCGTTTTTCGCAAATCTAATTTCAGCGAAACCACCAAAG

GGGGTGAGAATCGGATGTAAATTTTTTTCTCTACAATTTTCATATAGAATTCGTCTCAATCCGAGGTCGTATGCAAAAGTTATGGCTGTTTTACCGAACAACACTTTTTCTTGCACCCCGGCGCCCGGCAG

TAGATCCAATCTATCACCGTTGCGCATCACATGCGCTCGGCACTTGACCTAGGTTCGATTCGATCAATCTGATTGATCTCCATCTTGCCATGACTGGATTTACATGTATCACTTAACTCGGATGGCGGAAT

TCCGACCGGTACGAGTAGATCGTTACAAGACCAACACGGTAAAATCACGAACCATGATCAGAGACTCATCTACAACCGCGCATATCTCCATACGCACTCATCCGAACCTATAACAACGCAGTAACGGCT

CTGATACCACTGAAGGGTTATGAGGATGCTACGCTAGCCGGAAAACAAAAATTTCGACCTCATAAACCAGGATCTACTGCTAGTTATAGATCACGGGATTACCACTAGACGCGCAGGTGCAGCGGAAG

CGACTCGATGTAGTCGAACGCGTCGTAGCAGTGCCGTGCAGTTGCGAGGTCGTCCGTACGTCCGTCCCCTTCGTGCGGTTCGTCCTTGTGCTCCAGATGCAGCACCTCCGAGGTATCCACACGTACAGGG

AGGAAGCGTTGCGCCTCCGGACTGCTAGGTCCACGAGGAGCAGCAGGCGCGGGCGTAGGATGGGCGGCGGCGGCTGCCAAAAGGCATGGAATGCTTAGCCCCGTTGCCCACCCCACATATTTATAGGC

GTCCCTAATGAGCTCCCGAGTTGGAGGCCCATTAGTAACCCTAAGCCTTGTCTAACTCGGATCCAATCCGAATTAGGCTTCCAGCCCCTTAAGCGTGCGACCCTATGGGTTCACGCACACATAGACATGG

CCCGAGTACTCCTACTCGGCCATTAGTTGATAGCGGCCTCTAGCAAGGCATGTCAACTCCTATGCGTACGCAAAGATCATATCAGACGAACCACCACAAAACCACATACTTGTTATTCCCTTGCCTCACG

ATATTTGGTCCAACTCATAGGTTAACACTTAACCCAAGCACGGCCATGCATTTCTTGATCTAATCATTAGAGTGATCCAGTGATATCTCTCTCATATATAGAGAGGGGCAAATTCCATCTTGATTGTCTAT

GTCTCATAGCATGTTTCCCGACAAACCCGAAAACCACCTTTATAACTACCCTGTTACGGAGTAGCGTTTGATAGTCCCTGAGTAGGTCGTTTCACATCTTGAATACATACAACAATCTCAGGTCTAAGGA

CATAACGTACATGATGTGAATAGAGATAATAACGACATCTCACGTTGGGTCAGTCCAGCCCCATGTCATACATGTGCCCACATTATTAGTTTGACATCTCCATGTCTATGACTTGTGAAACATAGTCATC

AACTAATAATGTGCTGATCCATTATTCATGTGTGTCCTCACACGAACTCTGACCAGGGACAACATTAGAATAACCATACAAGTAAAAGAGTTTCACAAACAATTCACATAATTGCTAATCGATACAGGT

TGTCTTTAATGGAAATTCAATGAACTCATAATATATCATGGATACAAGGCAATATAATCATCTCTATGATTATCTCTAGGGCATACTCCCAACAATCTCCCACTTGCACTAGAGTTAATCTCGAAGATAT

CTAATACCCATAGCTCTTATGTGCGCCTCATGCTTAGGCTGTGGAAGAGCCTTTGTCAAAGGATCTGCAACATTCAAATCTGTGTGTATCTTGCACATCTTGATCTCATCTCGTCTAACGAACTCTCGAAT

GAGGTGAAATTTCCGCAGTACATGTTTGCTCTTTTGGTGATTCCTTGGCTCCTTAGCCTGCGCAATTGCCCCATTGTTGTCACAATGTAGATTCAATGGGCTGGACGCATTCGGAAACACACCAAGTTCAA

TGAGGAACCTCCTCATCCAAACACCTTCCTTCGCAGCTCCAGAAGCTGCAATGTACTCGGCCTCTGTTGTAGAATCGGTCACCGTCTCCTGCTTGGAACTTTTCCAACTTACAGCACCACCATTTATTGTG

AACACAAAACCTGATTGAGACTGTGAATCATCCGTGTCAGTTTGGAAGCTAGCATCGGTGTAACCATTTACAACGAGCTCCTCCTCACCTCCATAGATTAGGAACACATCTTTAGTCCTTCTCAAGTACT

TAAGAATGTTTTTAACAAGTGTCCAGTGACTCTCTCCTGGATCAGCTTGGTACCTGCTCGCAACACTTAGAGCATATGAGACATCTGGGCGAGTACATATCATGGCGTACATGATGGAACCAACTGCCGA

GGCATATGGAACCTTACTCATGCGCTTCCGCTCATCAGCTGTCGAAGGACACTGTTTATCGCTAAAGCGCATACCATGTGACATAGGCAAGAACCCTTTCTTGGACTGTTCCATGTTGAATCGTTTCAAC

ACCTTGTCAATGTACGTATCTTGGCTTAATCCTATAAGCCTCTTCGATCTATCTCTATAGATCTTGATGCCCAAAATGTATGCTGCTTCTCCTAAATCCTTCATAGAAAAATTATTATTCAGTGAAGTCTTT

ACGGATTGCAACATAGGAATGTCATTCCCAATCAATAATATGTCATCCACATACAAGATCAGAAATACAACAGCGCTCCCACTTTCCTTCTTGTAAACACAAGGTTCTTCTTCATTCTGACGGAAGCCAA

ACCCTTTGACCACTTCATCAAAACGAATGTTCCAACTCCGAGATGCTTGCTTTAACCCATAAATGGATTTCTGAAGCTTGCAAATTTTTCCAGCATTGTTTGGATCAACAAAACCTTCGGGCTGTATCATA

TACACGTCCTCATCCAAATTTCCATTTAGAAAGGCTGTTTTAACATCCATCTGCCATATCTCATAATCGAAATAAGC

MsSat185

CGCTCCGGCCCAAGCCTCCGGCGACGGGCCTCCGCTTCGCTCCGTCAGAAGCCCGGCCTATATCCTGGAGTGAATTTGGAACAACTCCAACAAACTCCACCTTGAGACAAATTCCTTCTTGTAGCATAAA
ACGAACCTTCACCCTGAAACAACAAAGAAATACTTCCGGTGCCAAATAGCCTCTTGGGCTAAACAGTTATACCAACTAAGCTTGAGCACAGCTCAAACTTAGCAACAGGAATAGGCTTTGTCATCATAT
CAGCAGGATTATCATGTGTGCTTATCTTGCATACCTTCAGCTTACCTTCTTCAATCACATCACGCACAAAATGATACTTGATATCTATGTGCTTAGTTCTCTCATGGAACATCTGATCTTTAGTGAGGTAA
ATTGCACTTTGACTGTCACAGTGCAAACTTATGCAAGATTCAACTCCACAAAGCTCAGCGTACAAACCTTTCAGCCAAATTAATTCTTTGCACGCTTCACAAATAGCCATATATTCTGCTTCAGTAGTAG
ACAAAGCAACAACTGACTGTAAAGTAGCCCTCCAACTCACAGCACAACCACCAATAGTGAACACATAACCTGTAAGTGATCTTCGCCTGTCCAGATCAGCAGCATAATCAGAATCCACATAGCCAATA
AGACCCTTATCAGTTCTTCCAAACTTTAAACAGGAATCTGCTGTGCCTCTGAGGTACCTGAAAATCCACTGAACTGCCCTCCAATGTTCTTTGCCAGGATTAGACATATATCTACTAACAAGACTCATAG
CATATGACAAATCTGGACGAGAGCAAACCATGGCATACATCAAAGAACCAACAGCACTAGAATATGGAACTCTTGACATGTACTCAAAATCCTCATCCGTACTAGCACATTGTAAAGCTGATAATTTGA
AATGAGGAGCAATAGGAGTACTAACAGACTTTGCATCATGCATGTTGAAACGCTGAAGAACTTTCTTAATGTAACTTTGCTGACTAAGAAATAACAAACCAGAATTTCTGTCTCTTGTAATTTCCATACC
TAGAATCTTCTTAGCAGCACCAAGATCCTTCATCTCAAACTCACTACTCAACAGTTTCTTCAACGTAGTGATTTCTTTCTTGCTCTTGGCAGCAATCAACATATCATCAACATATAACAGCAAATATATAG
GTGATCCATTAACATGCTTGATGTAAACACAGCTATCATACTTAGATCTTTTAAAACTGTGTGCTAACATAAATGAATCAAACCTCTTGTTCCACTGACGAGGGGACTGTTTCAAACCATACAAGGATCT
CTTCAACTTGCACACATATTTTTCCTTGCCAGGCACTATGAACCCTTCTGGTTGGTCCATGTATATGTCCTCCTCAAGCTCTCCATGCAAAAACGCAGTCTTCACATCTAACTGCTCAAGCTCAAGATCAT
GTGAAGCAACAATACTAAGGAAAGTACGAATTGAACTGTGTTTTACCACTGGAGAGAACACATCATTGTAGTCAACACCCGGAATTTGACTGTAGCCTTTAGCAACTAACCTTGCCTTATACTTTGGAG
GCTCGCTTGGAGATAAACCCTCCTTTCTCTTGAAGATCCATTTGCAGCTGATGGTCTTCTTATTCTTAGGCAAAGGTACAATCTCCCATGTACTGTTCTTCTCAAGAGACTGCATCTCCTCATGCATAGCA
GAAATCCACTTCTCGCGGTCACCAGAAACAACAGCTTCAGTGTACGTTGCTGGCTCATGAACGTTCTCCACCTGTTCAGCACAACTCAAAGCATAGTAAGACAAATTACACTCTTCAATAAGACGCTTAG
GAGGTGCAATTGTCCTTTTTGACTTGCGTTGAGCAATAGGTAAATCCTCCTCTAACTGCAAAATAGGAGGAGTTTGCTGAACATCATCATGAGCATCATCCTCAGCAACATCATTATCATGGTCACCATG
CTCATCAACAGGCTCCACCTGCACACCTGTATCATCATCAACATGCTCCACCTGCATGCGCATACGCTGCAGCTCTTTTTCTGGAACATGATCTGAGGGCAAACTGTCAGTAAACATCACAGATTCATTG
AAAACAACACTCCTGCTCATAAAAGTCTTTCTAGTTTCAGGATTCCATAATTTATATCCTTTAACTCCAGAACCATAACCAAGGAAAAGACACTTAACAGCTCTAGGCTCTAATTTTCCATTATCAACAT
GAGCATAAGCAGTGCATCCAAAAACTTTCAACTGTGAATAATCAGCAGGCATACCAGACCATACCTCAATGGGAGTTCTTTTATTTAGTGGAATAGAAGGCGACCTGTTTATCAAGTAACAGGCAGTAT
TAGCAGCTTCAGCCCAAAAATGCTTGCTCATCCTGGCATTGGACAGCATGCAACGGGCCCTCGAGATGATGGTTCGGTTCATGCGCTCAGCCACACCATTCTGCTGAGGAGTGTAGGGTATGGTGTGAT
GCCTAACAATGCCTTCCGATCTGCAATAATCATTAAATTCACGCGAACAAAATTCTCCACCATTATCAGTACGAAGCAATTTTACCTTCTTTTCAGTTTGCCTTTCTATCATAACTTTCCACTCTTTAAAAG
CAGCAAAAGTATCATCTTTATTTTTCAGAAAATAAGGCCAAACTTTTCTAGAGTAATCATCAATAATGGTAAGCATGTAACGAGCACCACCAAGTGAGGACTTACGGGAAGGACCCCATAAATCAGCAT
GAACATAATCAAGAGTACCTTTAGTGGTGTGAACAGAAGTGTTGAAATGTACCCTTTTATGCTTCCCGAAAATACAATGCTCACAAAACTTTATTTTACTCGAAGTGCAGCCATCCAGCAGGTTTCTCTT
CATCAATTCTGCCATACCATGGTGACTCATATGTCCCAAACGCATATGCCAAAGGTTAGTTTTACTAGGTTCATCATTAGTAACAGCAGCAACAGCGGAATCAGAACCAGGTAAGGTACACCCTCTAAG
GACATATAACTTTGCAGAATTCATATCACCTATCATATGAATGAGAGAACCTTTTGATACCTTACACACTCCATCTGAACCGGAATATTTGTAACCTTCTGCATCAAGCGTGCTCAGGGAGATAAGATTT
CTGGCCATCCCTGGTATGTGTCTCACATCTTTCAGTGTGCGTATCATGCCATCATGCATCTTGATCTGAACTGATCCAATCCCCACAATGTCACATGGGTTATCATCTCCCATACGCACAACATCTCCACT
CTGCACAGACTTATACGAGCTAAACCAATTTCTGTTAGTGCAAATATGAAATGAACATGCGGAATCGAGAATCCATTCATCATGACCAGCAACACAACCAGCAAATACTACCAGACAATCTCCAGAATC

ATCATCAGACGCAGCAGCAGCAACAGAGACCTTACCAGCCGACTTGTGTTTCCTCTTCTCCTTATTCTGTACTTTTCTGCAATCCTCAACATTATGATTTGTCAGCTTGCAATAAACACAGAACTTTTTAT
TACCATGCGGCTTGGACTTTGAACGGCCTCTCCTTTCGTAGTTCTTTCTACCATCATTGCCATCATTGGAGGATCTCTTCTCAGTTCTGCCTCTCACATGCAAAGCATCGCCCTTGGAGGACGACGTCCCG
TCATTCTGCACCATGCCTCTCATCTTCTCCCTAGACTGGAGAGCCTCATAAACTTCCTTTAGGGTTAGTTCATCACGGCTTAATAGTATGGTATCTCGAAAATTTGCAAACGAATTAGGCAAAGAACATA
GCAGTAAAAGACCTAAGTCCTCATCATCATACTTCACCTCCATCGACACTAGGTCGGCGACGATCTTCTTGAATTCAGCGAAGTGGCTCATCACCGAACCACCTTCTTGCAGCTTCAGCGTGAACAACTT
CATCTTCATCTGCATCTTACTGGTTAGATCCTTGGACATACAGATCGATTCCAGTTTCAGCCATAGTTCTGCAGCAGTTTTCTCTTTCAGACACTCCTGCAAAATATCATTATGAAGATGCAACTGAATTA
GGGCGAGCGCCTTCTGATCCTTGCGGATCTCTTCTGGAGTCCACTCGGCCTTCCTCTTTCCGAAACTATCCAGCGCCTCATCATAGTCATGAGTCTGCGCCAGAATCCCCCGCATCTTGACTTGCCATAGC
GAGAACCGCGTGTCGTAGTTCAGCAGCGGAAGATCGAACTTCATCGACGTCGTCATGAACCCTAACAGCAACCAAAGCTCTGGTACCACTTGTTATGAACGACGTATAGGAATATGAAGTAAAGAATC
AAGCCACAGAGGAGACACACGATTTAACGTGGAAAACCCCTCCGATGTGAAGGGGAAAAACCACGGGCACCAGCCAGCAACAATCTCACTATCTCAAGAGTTTTGGGTTACACGCCTATGGCGGCTTA
CAAGAGAATCAAGTCTCCACGAAACCCTA

MsSat192	CAAAAAAGGGAATGCAACACGAGGACTTCCCAGGAGGTCACCCATCCTAGTACTACTCTCGCCCAAGCACGCTTAACTGCGGAGTTCTGATGGGATCCGGTGCTTTAGTGCTGGTATGATCGCATCCGA<br>CATACCCTGCGTCCTCCTTCCTTTTATGCCCGCGCCCCCGCGTCCGGCCCGCCCGTGGCTTCGGCGCGCCGGTTCCCCTTCCGCCTCCCCCCTCACCTGGACGGTGCCGCCTAGGAAAACGAAGAAAATA<br>TGACGAAGGGACGGCCAGAAAACAGTTTTTGGAAAAAAAAA

Table S2. The information of representative candidate variation satellite sequence from second cluster.

Name	Sequence information (5’ - 3’)
Ms1-A	CACCTAGGAGTTCCATCGGGTGCGTCCAAAATGATTTCTGAGCCTATGGTACGTTCGGCGCAAACCATGCACCTATCTTGCGTCAAGATTAGCACTATCTCCAAACGGACCAAAACGAGCTTCCACTTGAGCCTCGT
Ms1-B	ACGTGGGTCATGCAGAATCTCACTTGTTTCCGTTTGGAGACAGTGTTAGCGTCGGTGCAAGATAGGTGCATGGTTTGTGCTAGACATACCATAGGCTTAGAAATCATTTTGGACGCACTTGATGTTACCACTAGGTG
Ms1-C	ACACGGGTCATGTCGAATCACGTTTCGGTTCGTTTGGAGATGGTGTTAGTGTCGGTGCAAGATAGGTGCATGGTTTGCGCCAAACGTACCATAGGCCCAGAAACCGTTTTGGATGCACCGATGGTACTCCTAGGTGG
Ms1-D	ACGAAGCTCAACTGGAAGCTCGTTTTGGTCCGTTTGGAGATGGTGTTAGTGTTGATGCAAGATAGGTGCACGGTTTGCGCCAAACGTACCATAGGCTCAGATATCGTTTTGGACGCACCCGATGGAACTCCTAGGTG
Ms1-E	ACGTGGGTCAAGTGGAATCTCGTTTCGGTCTGTTGGGAGACAGTGTTAGTGTCGGTGCAAGATAGGTGCACGGTTGGCGCCGAACATACCATAGGCTCGGAAATCATTTTGGACGCACCCGATGGTACTCCTAGGTG
Ms1-F	ACGAGGCTCAAGTGGAATCTTGTTTCGGTCCGTTTGGAGATAGTGTTAGTATTAGTGCAAGATAGGTGCACGGTTTGTTCCAAACAAGGCTCGGAAATCATTTTGACCGCACCCGATAGAACTCCTAGGTG
Ms1-G	ACGTGGGTCAAGTGGAGGCTTGTTTCAGTCAGTTTGGAGATAGTGTTAGCCTCGACGCAAGATAGGTGCACGATTTGCGCCAAACGCACCATAGGCTCAGAAATCAGTTTGGATGCACCTGATGGTACTCCTAGGTG
Ms1-H	ACGAGGCTCAAGTGGAATCTCGTTTCGGTCCGTTTGGATATAGTGTTAGTATTAGTGCAAGATAGGTGCACGGTTTGTGCCAAACGTACCATAGGCTCAGAAATCGTTTTGGACGCACCCGATGGTACTCCTAGGTG
Ms1-I	ACATGGGTCATGTGGAATCTCACTTCTTTCCGTTTGGAGACAGTGTAAGTGTCGGTGCAAGATAGGTGCACATTTTGCGCCAAACGTACCATCGGCTCAGAAATCGCTTTGGACGCACCCGATGGTACTCCTAGGTG
Ms1-J	CACCTATGAGTTCCAACAGGTGCGCCCAAAATGACTTCCGAGCCTATTGCACGTTCGGTTCAAACCGTGCACCTATCTTGCGTCGAGACTAACACTATCTACAAACGGACTGAAACGAGCCTCCCCTTGACCCACGT
Ms1-K	ACGTGGGTCATGTGGAATCTTATTTCGGTCCATTTGCAGACAGTGTTAGATAGGTGCACGGTTTGCGCCAAACATACCATAGGCTTAGAAATCATCTTGGACGCACCCGATGGTACTCCTAGGTG
Ms1-L	ACGTGGGTGAAGTGGAAGCTCGTTTCAGTCCATTTGGAGATAGTGTTAATCTCGACGCAAGATAGGTGAACAGTTTGCGCTGAATGTACCACGGGCATGGAAATCGTTTTGGACGTACTCGATGGTACTCTTGGGTG
Ms1-M	CACCTAGGAGTTCTATCGGGTGTGTCCAAAACGACTTCCAAGCATATGGTACGTTCGGCATAAACCTTGCACCTATCTTGCGTCAGGATTAGCACTATCTCCAAATGGACCAAATCAAGCTTCCACTTGAGCCTCGT

Table S3. The PCR primers used for development of FISH probes for representative candidate variation satellite sequence.

Probe name	Primer name	Primer sequence（5’- 3’）
Ms1-A	Ms1-A -F	TTGCGTCAAGATTAGCACTATCTCCAAACG
	Ms1-A -R	GAAGCTCGTTTTGGTCCGTTTGGAGATAGT
Ms1-B	Ms1-B-F	TTGCACCGACGCTAACACTGTCTCCAAACG
	Ms1-B-R	GAATCTCACTTGTTTCCGTTTGGAGACAGT
Ms1-C	Ms1-C-F	TTGCACCGACACTAACACCATCTCCAAACG
	Ms1-C-R	GAATCACGTTTCGGTTCGTTTGGAGATGGT
Ms1-D	Ms1-D-F	TTGCATCAACACTAACACCATCTCCAAACG
	Ms1-D-R	GAAGCTCGTTTTGGTCCGTTTGGAGATGGT
Ms1-E	Ms1-E-F	TTGCACCGACACTAACACTGTCTCCCAACA
	Ms1-E-R	GAATCTCGTTTCGGTCTGTTGGGAGACAGT
Ms1-F	Ms1-F-F	TTGCACTAATACTAACACTATCTCCAAACG
	Ms1-F-R	GAATCTTGTTTCGGTCCGTTTGGAGATAGT
Ms1-G	Ms1-G-F	TTGCGTCGAGGCTAACACTATCTCCAAACT
	Ms1-G-R	GAGGCTTGTTTCAGTCAGTTTGGAGATAGT
Ms1-H	Ms1-H-F	TTGCACTAATACTAACACTATATCCAAACG
	Ms1-H-R	GAATCTCGTTTCGGTCCGTTTGGATATAGT
Ms1-I	Ms1-I-F	TTGCACCGACACTTACACTGTCTCCAAACG
	Ms1-I-R	GAATCTCACTTCTTTCCGTTTGGAGACAGT
Ms1-J	Ms1-J-F	TTGCGTCGAGACTAACACTATCTACAAACG
	Ms1-J-R	GAGGCTCGTTTCAGTCCGTTTGTAGATAGT
Ms1-K	Ms1-K-F	TAACACTGTCTGCAAATGGACCGA
	Ms1-K-R	GAATCTTATTTCGGTCCATTTGCA
Ms1-L	Ms1-L-F	TTGCGTCGAGATTAACACTATCTCCAAATG
	Ms1-L-R	GAAGCTCGTTTCAGTCCATTTGGAGATAGT
Ms1-M	Ms1-L-M	TTGCGTCAGGATTAGCACTATCTCCAAATG
	Ms1-L-M	GAAGCTTGATTTGGTCCATTTGGAGATAGT
